# The dynamic genomes of *Hydra* and the anciently active repeat complement of animal chromosomes

**DOI:** 10.1186/s13059-025-03653-z

**Published:** 2025-07-01

**Authors:** Koto Kon-Nanjo, Tetsuo Kon, Tracy Chih-Ting Koubkova Yu, Diego Rodriguez-Terrones, Francisco Falcon, Daniel E. Martínez, Robert E. Steele, Elly Margaret Tanaka, Thomas W. Holstein, Oleg Simakov

**Affiliations:** 1 Department of Neurosciences and Developmental Biology, University of Vienna, Vienna, 1030 Austria; 2 Molecular Evolution & Genomics, Centre for Organismal Studies, Heidelberg University, Heidelberg, 69120 Germany; 3 Institute of Molecular Biotechnology, Vienna, 1030 Austria; 4 Vienna BioCenter PhD Program, Doctoral School of the University of Vienna and Medical University of Vienna, Vienna, 1030 Austria; 5 Department of Biology, Pomona College, Claremont, CA 91711 USA; 6 Department of Biological Chemistry, University of California, Irvine, Irvine, CA 92697-1700 USA

**Keywords:** Telomere-to-telomere genome assembly, Anciently active transposable elements, Genome expansion, Stem-cell genomes, Hydra

## Abstract

**Background:**

Many metazoan genomes are characterized by highly conserved chromosomal homologies that predate the ancient origin of this clade. This conservation has been tested by expansions of selfish DNA elements, in particular transposable elements (TEs). While comparative genomics studies have highlighted their diversity across animal genomes, common principles underlying their evolution along deeply conserved chromosomes have been elusive. A detailed mechanistic understanding from phylogenetically key and early branching animal species has been lacking.

**Results:**

We present a comprehensive stem-cell resolved genomic and transcriptomic study of the freshwater cnidarian *Hydra*, an animal characterized by its high regenerative capacity, the ability to propagate clonally, and an apparent lack of aging. Using single-haplotype telomere-to-telomere genome assemblies of two recently diverged strains and utilizing unique features of hydra biology allowed us to sequence and compare the individual genomes of hydra’s three stem cell lineages. We show that distinct TE families are active at both transcriptional and genomic levels via non-random insertions in each of these lineages. We show that the core set of these active TE families, primarily composed of DNA elements, is evolutionarily deeply conserved and contributes to consistent genomic expansions in metazoan lineages. These anciently active TEs differentially contribute to structural variants around loci associated with cell proliferation and long-range topological contacts. This is in strong contrast to the frequently observed and highly varied substantial genome expansions that often happen via retroelements.

**Conclusions:**

Our study suggests an ancient and conserved role for these core TEs as self-renewing components of animal chromosomes.

**Supplementary Information:**

The online version contains supplementary material available at 10.1186/s13059-025-03653-z.

## Background

Animal genomes have been shown to be remarkably conserved at both chromosomal [1, 2] and sub-chromosomal levels [3–5]. While this remarkable conservation can be explained by gamete balancing issues that can arise in the case of inter-chromosomal translocations in the germline [6], the evolutionary forces that keep regulatory landscapes intact within chromosomes are less well understood.

One such force is the continuous expansion and contraction of chromosomes through the action of transposable elements (TEs). How this dynamic chromosomal landscape may contribute to macroevolution, and the resulting evolutionary constraints on chromosomal elements themselves, is unclear. Many cases of TEs contributing to genome expansions, changes in expression levels, regulatory element evolution, and structural variations have been reported (Additional file 1: Supplementary Note 1 [1, 7–40]). In animals, class I (copy-and-paste propagating TEs) have been especially strong contributors in many reported genome expansions [18, 41] (Additional file 1: Supplementary Note 1). The role of class II (cut-and-paste DNA elements) has been elusive and their broad contribution to genomic expansion remains unclear [18]. Recent studies also highlight a decoupling between repetitive element accumulation and chromosomal conservation (synteny), with extremely large animal genomes showing conserved synteny and few rearrangements [42]. To find common evolutionary and functional principles behind these genomic trends requires telomere-to-telomere haplotype-resolved genome assemblies and a detailed understanding of TE activity and insertion dynamics for key early branching animal species. Very few such resources are available.

The freshwater cnidarian *Hydra* sp. is a powerful and unique system for addressing these questions. As a member of the sister phylum to bilaterians, hydra occupies an important evolutionary position, spanning the earliest animal origin and diversification at least more than 550 million years ago based on fossils and molecular evolutionary analysis [43, 44]. Hydra was the first animal in which whole body regeneration was demonstrated [45], and the first animal in which a developmental organizer was identified [46–48]. Hydra serves as a simple yet powerful model system, providing profound insights into stem cell dynamics [49]. Adult polyps of *H. vulgaris* do not show signs of ageing [50, 51]. Homeostasis and the extensive regeneration capacity of hydra are maintained by three stem cell lineages, endodermal epithelial, ectodermal epithelial, and interstitial/germline stem cells (i-cells). Endodermal and ectodermal epithelial stem cells are unipotent, only producing epithelial cells. I-cell stem cells are multipotent and produce nematocytes, nerve cells, gland cells, and germline cells [52], though the rate may depend on the developmental stage [53]. The three stem cell lineages maintain their distinct identities and do not interconvert. Hydra regeneration from bisected animals, small tissue fragments, and even aggregates of cells has been extensively studied since the first experiments by Abraham Trembley in the 1700 s [45]. More recently, transcriptomic studies have revealed the diversity of the molecular pathways that are active during hydra regeneration [32]. Comprehensive profiling of gene expression in each cell type and stem cell differentiation trajectories have been reported for the whole polyp of hydra through single-cell RNA-seq (scRNA-seq) [54]. While conserved components such as the Wnt signaling pathway have been investigated in developmental and regeneration processes in hydra, many species-specific genes and, particularly, TEs are expressed, with unclear functional or evolutionary impact [32, 55, 56].

The ability of hydra to propagate asexually has striking implications for its biology. If hydras are cultured by budding for multiple generations, mutations accumulate in each stem cell lineage independently [57]. Therefore, clonally propagated hydras are expected to have three distinct genomes (ectodermal, endodermal, and i-cell stem cell lineages). If TEs have diverging functions in each of the three genomes, we expect to find differences in their cell-type specific transcriptional activity and genomic insertion profiles. These profiles may suggest potential impacts on cellular function and adaptation.

The genus *Hydra* sp. is composed of two major evolutionary lineages, the symbiotic green hydra (*H. viridissima*) and the species-rich brown hydras (e.g., *H. vulgaris* and *H. oligactis*) [58]. These two lineages diverged 46–61 million years ago [58], yet in this relatively short evolutionary time-span, brown hydra genomes have experienced a tripling in genome size [36, 40] while still preserving the ancient animal chromosomal homologies [1]. The green hydra genome is similar in size to genomes of most other cnidarians (less than 500 Mbp) [59]; the brown hydras have some of the largest cnidarian genomes (around 1 Gbp or more), with over 60% of the total sequence comprised repetitive sequences, including TEs [36, 60].

Within the brown hydras, two *Hydra vulgaris* strains, AEP and 105, have been extensively utilized in molecular studies. The strain AEP, from North America [61, 62], routinely undergoes sexual reproduction in laboratory conditions and is widely used to create transgenic lines [63]. Conversely, the strain 105, which was collected in Japan (described as *Hydra magnipapillata* strain 105), has been maintained through asexual reproduction in the laboratory since 1973 [64]. Although it almost certainly engaged in sexual reproduction in its natural environment, the presence of sexually mature individuals in the laboratory is extremely rare.

Together, these properties make hydra a valuable model system to investigate ancient evolutionary trends of TE composition and function in the animal kingdom. To uncover the role of the evolving TE landscape in hydra stem cell genome(s) and its impact on shaping the otherwise preserved, yet expanded, ancient animal chromosomal elements, we present the haplotype-resolved telomere-to-telomere genome assemblies of *Hydra vulgaris* 105 and AEP strains and reveal different TE evolutionary expansions among the three stem cell lineages. Using fluorescence-activated cell sorting (FACS) followed by genome sequencing, we reveal distinct TE insertion profiles in the three stem cell lineages in AEP-background strains. We show evidence indicating how these TE families impact the genome and their neighboring genes. We provide direct observation that the three stem cell genomes of hydra undergo distinct and non-random changes and suggest this as a key structural role of TEs in the evolutionary adaptations of these stem cells. Finally, through multi-species comparisons, we find remarkably similar TE signatures across eukaryotes and metazoans, identifying core sets of a dozen TE elements, mostly comprised DNA transposons, that have consistently shaped chromosomal evolution for hundreds of millions of years.

## Results

### Haplotype-resolved telomere-to-telomere reference genome assemblies for 105 and AEP strains of *H. vulgaris*

**Fig. 1:**
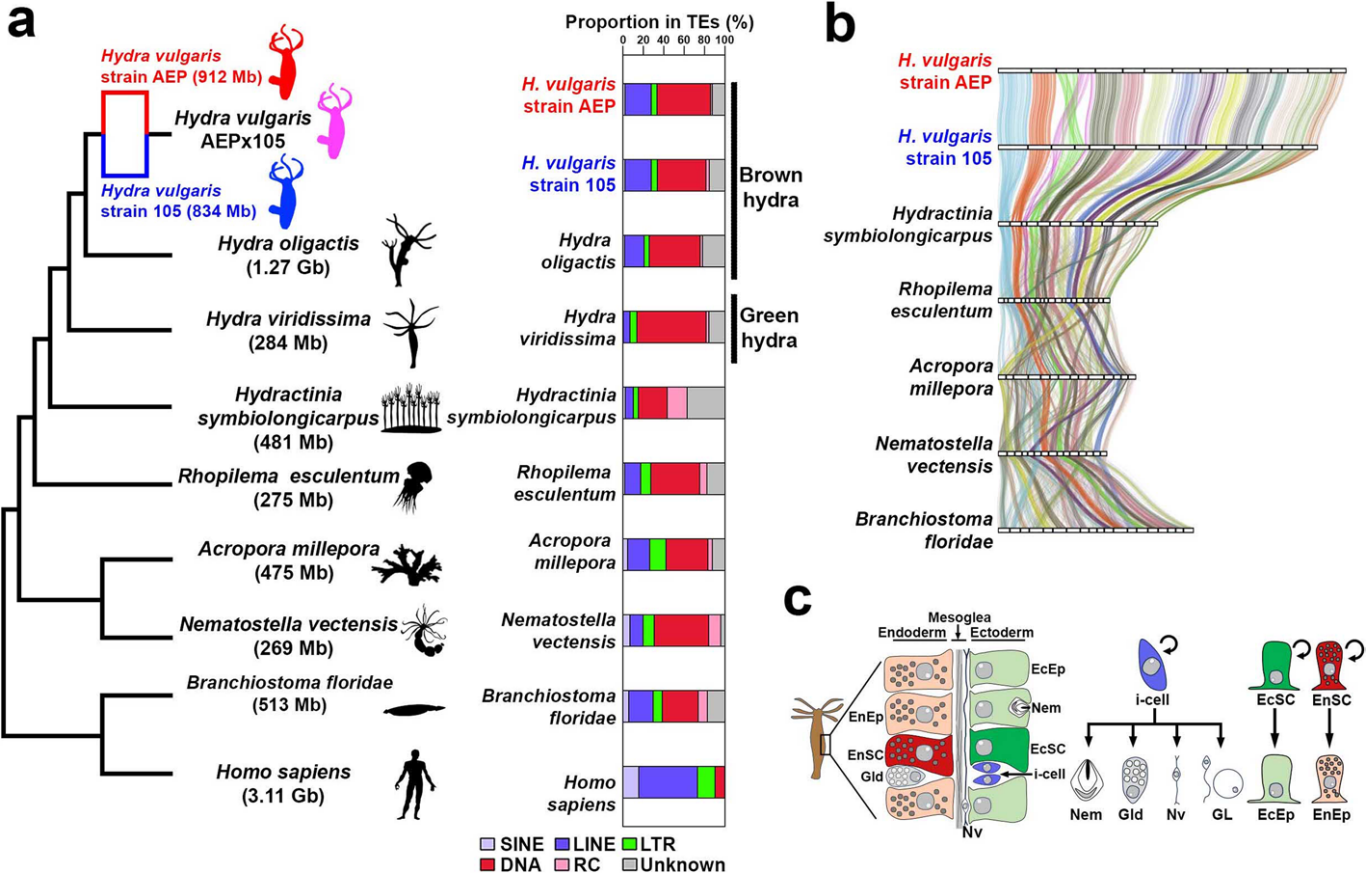
Haplotype-resolved telomere-to-telomere genome assemblies for 105 and AEP strains of *H. vulgaris* highlighting genome expansion. **a** Cladogram representing major cnidarian species. Numbers in parentheses indicate the sizes of publicly available genome assemblies. Animal silhouettes are from PhyloPic (www.phylopic.org). The bar plot represents the proportions of TEs relative to the total repetitive elements in the various genomes. **b** Comparative genomic synteny. The coordinates of 2440 single-copy orthologs from 6 cnidarians and one bilaterian (*Branchiostoma floridae*) are connected by lines. **c** The stem-cell system of hydra. Abbreviations: i-cell, interstitial stem cells; EcSC, ectodermal stem cell; EnSC, endodermal stem cell; Nem, nematocyte; Gld, gland cell; Nv, nerve cell; GL, germline cell; EcEp, ectodermal epithelial cell; EnEp, endodermal epithelial cell

While chromosomal-scale assemblies of *H. vulgaris* strain 105 and strain AEP have been published [1, 60], they lacked the telomere-to-telomere completeness and haplotype resolution needed to study the action of TEs in a stem cell lineage resolved manner (Fig. 1a–c). We utilized newly generated chromosomal conformational capture data from a AEP × 105 F1 hybrid (Methods) and new long-read sequencing to generate telomere-to-telomere assemblies (Fig. 1a, b, Additional file 1: Fig. S1a–h, Additional file 2: Tables S1–S3). As the sequencing data came from a hybrid of two recently diverged hydra strains (Fig. 1a, Additional file 1: Fig. S1a), our data produced a single haplotype for each of the strains with telomere-to-telomere quality (Fig. 1a, b, Additional file 1: Figs. S1b–h, S2a, b). Both strains showed a high degree of local (micro-) synteny conservation and the previously reported conservation of ancient animal chromosomal elements (Fig. 1b) [1]. However, the 105 strain genome showed a single apomorphic translocation between chromosome 5 and chromosome 15, which was previously suggested [1] and can now be confirmed with our data (Fig. 1b). Our analysis further identifies this event as a Robertsonian translocation where chromosome arms are exchanged at the centromeric regions (Fig. 1b, Additional file 1: Fig. S2c–e).

**Fig. 2:**
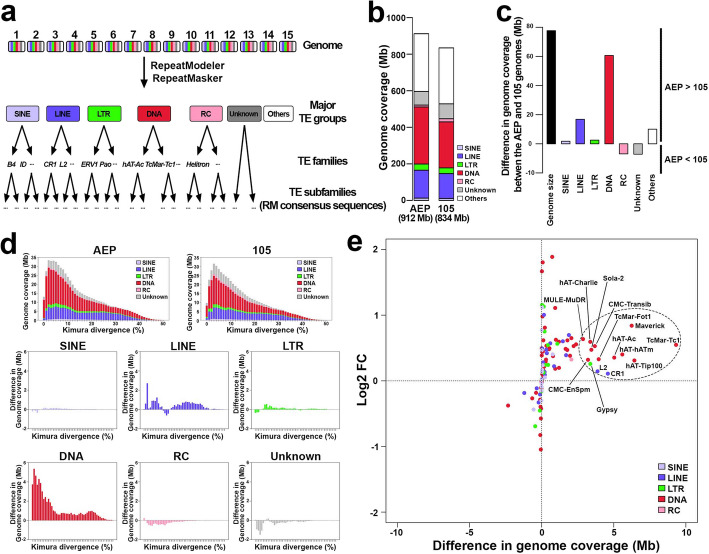
DNA transposons as the major contributor behind the recent AEP genome expansion. **a** TE classification pipeline and levels of TE annotation. Abbreviation: RM, RepeatModeler. **b** TE genome coverage in the AEP and 105 genomes. **c** Difference in TE genome coverage between the AEP and 105 genomes. **d** Kimura divergence and TE genome coverage. The top two panels show Kimura divergence and TE genome coverage for the AEP and 105 genomes. The bottom six panels represent Kimura divergence and difference in TE genome coverage between the AEP and 105 genomes, showing enrichment and recent insertions of mostly DNA elements, along with LINE and LTR elements, in the AEP strain. The scale of the x-axis in the bottom six panels is the same as that in the top two panels. **e** Difference and log2 fold change in TE genome coverage between the AEP and 105 genomes. The x-axis represents the difference in genome coverage of TE families between the AEP and 105 genomes (in Mb), while the y-axis displays the log2-transformed fold change (Log2 FC) in genome coverage of TE families between the two genomes. The dotted ellipse highlights a cluster of 14 TE families with over 2.5 Mb greater genome coverage in the AEP genome compared to the 105 genome (A-TEs)

When comparing the 13 chromosomes, excluding the chromosomes that underwent the Robertsonian translocation, between each haplotype genome, 12 chromosomes of the AEP haplotype were longer than those of the 105 haplotype suggesting that the AEP genome has experienced a genome-wide size increase (Fig. 1b, Additional file 1: Fig. S1e). We found that the major groups of TEs (including SINE, LINE, LTR, DNA, RC; Fig. 2a) accounted for 65.3% (596 Mb) of the AEP genome and 63.3% (528 Mb) of the 105 genome (Fig. 2b, c). Among these groups, DNA transposons were the most common, comprising 34.2% and 30.1%, followed by LINEs at 16.7% and 16.3% in the AEP and 105 genomes, respectively (Fig. 2b, c). When comparing the age distribution (Kimura substitution levels) among each major TE group between the AEP and the 105 haplotypes, we found that relatively young DNA elements and LINEs have increased in AEP, suggesting their contribution to the genome size increase after the divergence from the last common ancestor of AEP and 105 (Fig. 2d).

We used these genome assemblies to compare the two strains in detail and to dissect the repeat composition along the individual chromosomes. We note that automatic TE family annotation is prone to many genomic and bioinformatic artifacts [65]; therefore, we cross-validated our annotation using other available annotation databases, including Dfam [66], Repbase [67], and Gypsy Database [68] (Additional file 1: Supplementary Note 2 [67, 68]). While classification and the level of manual curation vary among these resources, we found generally consistent result of 106 uniquely assigned and annotated TE families present in the hydra genome (Additional file 2: Table S4), similar to the previously published estimates [36, 40]. The genome coverage difference of TEs between the AEP and 105 haplotype genomes is 67.6 Mb, which accounts for 87.1% of the genome size difference between the two genomes (Fig. 2c).

Using this detailed annotation, we identified the putatively active TEs (“A-TEs”) composed of 14 TE families with the enrichment in AEP relative to 105 (Fig. 2e, Additional file 1: Fig. S3, Additional file 2: Table S4, Additional file 1: Supplementary Note 2). These included 11 DNA elements (hAT-Ac, hAT-Charlie, hAT-hATm, hAT-Tip100, TcMar-Tc1, TcMar-Fot1, Maverick, Sola-2, MULE-MuDR, CMC-EnSpm, and CMC-Transib), two LINE elements (CR1 and L2), and one LTR element (Gypsy superfamily). These elements were found to contribute the most to the AEP genome size increase (Fig. 2e, Additional file 2: Table S4). A-TEs accounted for 36.6% (334 Mb) of the AEP genome and 35.8% (299 Mb) of the 105 genome, while non-A-TEs accounted for 28.7% (262 Mb) of the AEP genome and 27.5% (229 Mb) of the 105 genome (Additional file 2: Table S4). Although the genome size increase attributable to the non-A-TEs (93 families, 1.2%) collectively was slightly higher than that of the A-TEs (14 families, 0.8%), the per-family contribution of each non-A-TE family was substantially smaller compared to that of the active TE families. While the 14 A-TEs collectively affect whole chromosomes, some subfamilies of A-TEs showed local propagation in specific chromosomal regions in AEP characterized by the lack of local synteny, suggesting expansion through tandem duplication (Additional file 1: Fig. S4a, b).

This analysis, based on complete and haplotype-resolved assemblies, highlights a diverse set of putatively active TE (A-TE) families in the hydra genome, which suggests a more complex picture of the brown hydra genome expansion than the previous finding of a single retroelement contribution to the genome size increase in this clade [36, 40]. We next sought to investigate these A-TEs transcriptionally.

### Expression activity of transposable elements in hydra

**Fig. 3:**
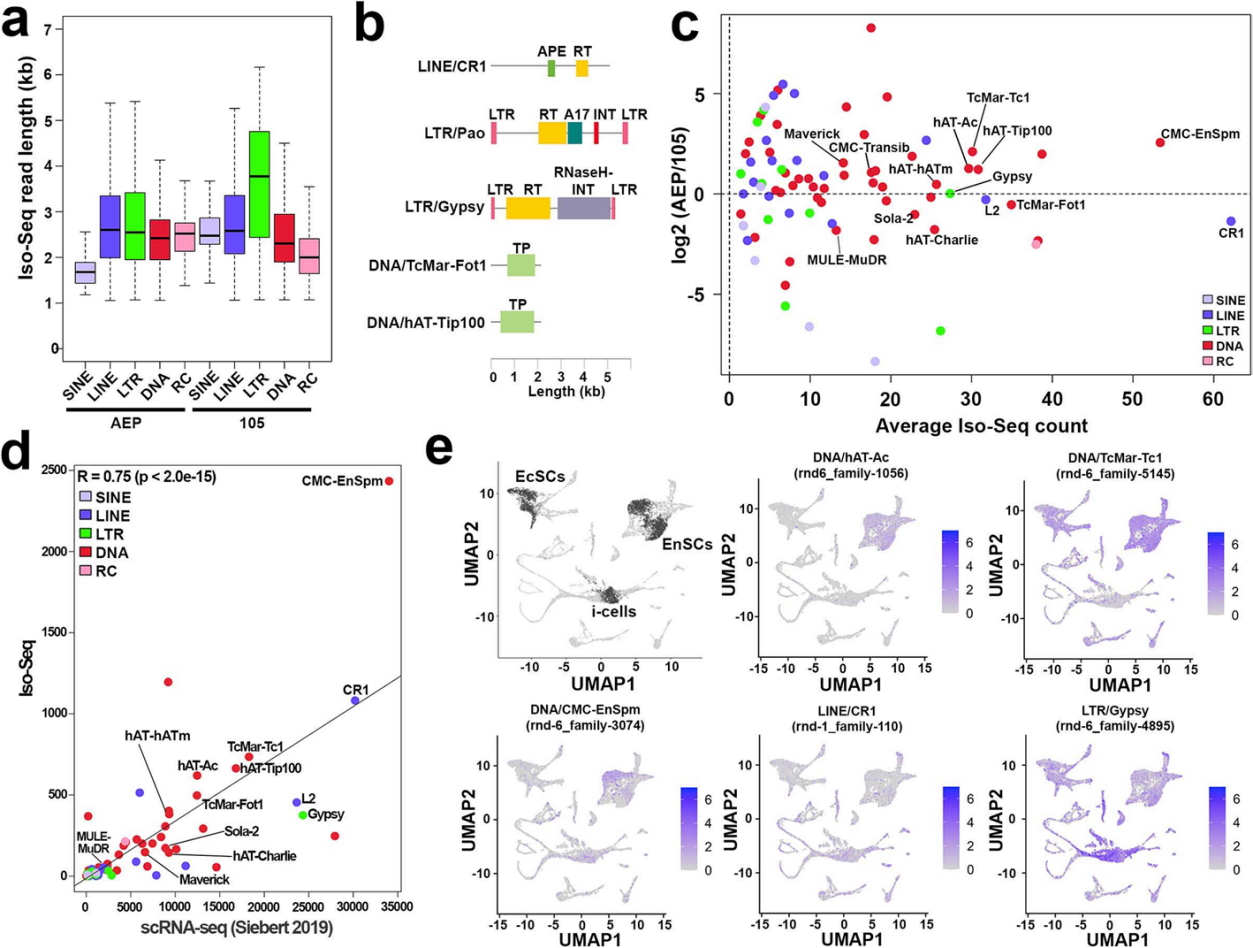
Strain and cell-type specific expression dynamics of hydra TE families. **a** The lengths of Iso-Seq reads capturing TE sequences. The boundaries of the box depict the first (25%) and third (75%) quartiles, while the median is denoted by the central horizontal line within the box. The whiskers extending from the box indicate the lowest and highest observed values. **b** Schematic domain structures of TEs identified by the Iso-Seq analysis. Abbreviations: APE, apurinic endonuclease; LTR, long terminal repeat; RT, reverse transcriptase; A17, peptidase A17; INT, integrase; RNaseH, ribonuclease H; TP, transposase. **c** TE expression difference between AEP and 105 strain. The x-axis represents the average transposable element (TE) expression between the AEP and 105 strains, while the y-axis displays the Log2 fold change in TE expression between them. **d** Correlation between Iso-Seq data from the present study and publicly available data. The x-axis represents the average transposable element (TE) expression in publicly available scRNA-seq data [54], while the y-axis shows the average TE expression in the Iso-Seq data from the present study. **e** TE expression profile at the cell-type level. In the left upper panel, stem cell populations are colored in black and other differentiated cells colored in gray. The rest of panels show representative plots of A-TE subfamily expressions

While expression of TEs has been proposed in hydra in previous studies [32, 69], validation using different sequencing technologies has been lacking. To identify a high confidence set of expressed TEs, we analyzed an Iso-Seq transcriptome generated from whole polyps of the 105 × AEP hybrid line. We identified 29,833 transcripts (0.32%) derived from TEs containing at least 1 kb of annotated sequence, including 14,302 transcripts (0.15%) from the AEP haplotype genome and 15,531 transcripts (0.16%) from the 105 haplotype genome. These transcripts included the major groups of TEs (Fig. 3a, b) and encompassed 86 different TE families (75 families and 73 families for the AEP and 105 haplotype genomes, respectively) (Fig. 3c). The average transcript length suggests that near full-length transposon sequences are being captured by Iso-Seq, with LTRs exhibiting the highest average length of 3.5 kb in the 105 haplotype genome (Fig. 3a). We also find that 30.9% of TE Iso-Seq reads in AEP and 29.7% of TE Iso-Seq reads in the 105 strain have trans-spliced leader (SL) sequences [36, 70] at the 5′ end of the reads. For Iso-Seq reads of non-TEs, 48.1% were found to have SL sequences. These sequences are associated with a reaction known as spliced leader trans-splicing, in which SL RNAs are added to the 5′ end of mRNAs through a trans-splicing mechanism [71]. This suggests that transcripts of TEs are utilizing hydra’s endogenous trans-splicing mechanism (Additional file 1: Fig. S5a, b), similar to the protein-coding genes [36]. All previously identified putatively active TEs (A-TEs) showed expression (Fig. 3c, Additional file 1: Fig. S5c, d, Additional file 1: Supplementary Note 2). Of all A-TEs, 11 A-TEs, excluding CMC-Transib, Maverick, and MULE-MuDR, were among the top 20% of the 86 TE types according to the expression level (Fig. 3c). Thirty-three percent of TE Iso-Seq reads were mapped to intronic regions, 66% of TE Iso-Seq reads were mapped to intergenic regions, and less than 1% were mapped to exons, across over 4087 and 4672 loci in the 105 and AEP genomes, respectively (Additional file 1: Fig. S5c, e).

Next, we analyzed the expression profiles of TEs using the publicly available single-cell RNA-seq dataset from adult polyps of the AEP strain [54]. We quantified the expression levels of TEs that had been confirmed to be expressed by Iso-Seq in the whole polyp (Fig. 3a–d). A significant correlation was observed between the expression levels of TEs in Iso-Seq and scRNA-seq (*R* = 0.75, *p* < 2.0e − 15; Fig. 3d). We investigated TEs that showed significantly higher expression levels in one or more of the six cell populations—comprising interstitial stem cells, cells differentiated from interstitial stem cells, ectodermal stem cells, cells differentiated from ectodermal stem cells, endodermal stem cells, and cells differentiated from endodermal stem cells—compared to the rest of the cell populations (Fig. 3e, left upper panel). At the family level, six TEs (TcMar-Tc1, CMC-Chapaev-3, PIF-Harbinger, hAT-Charlie, hAT-hAT5, and MULE-NOF) showed specific expression in one or more cell populations, with TcMar-Tc1 and hAT-Charlie classified as A-TEs (Additional file 2: Table S5). All A-TEs were expressed at least in i-cells which is consistent with the fact that i-cells can give rise to germline cells [53], whose genomes are inherited by all three stem cell lineages through sexual reproduction (Additional file 1: Figs. S5d, S6, Additional file 2: Tables S6, S7, Additional file 1: Supplementary Note 2).

In summary, these data provide a comprehensive bulk, tissue, and cell-type resolved cross-validation analysis of hydra TE expression dynamics, revealing distinct and cell-type specific patterns of TE expression and the high expression activity of the A-TE families.

### Identification of stem cell-specific transposon insertion sites using long-read sequencing

**Fig. 4:**
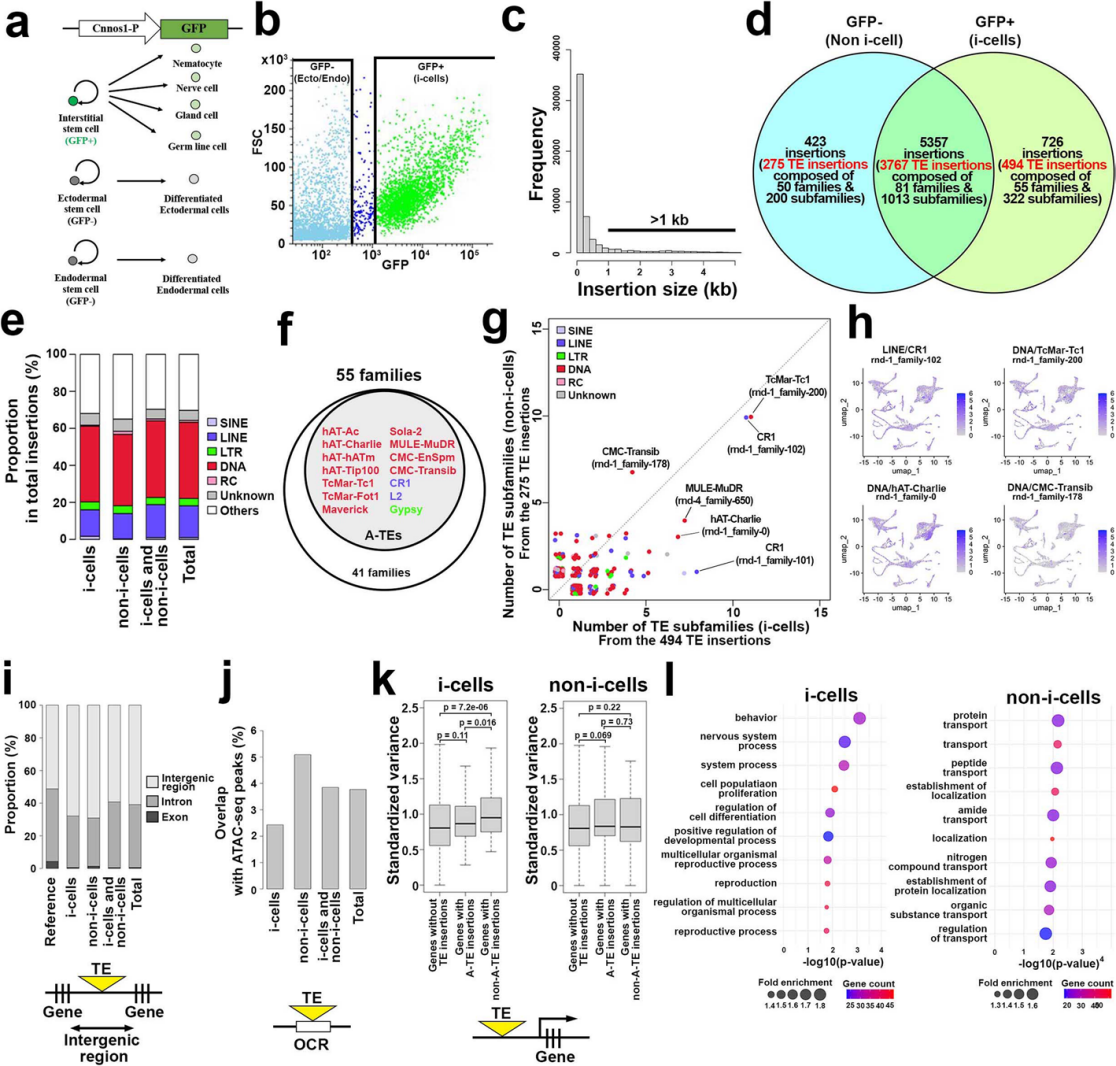
TE insertion dynamics of stem cells in hydra. **a** Cnnos1-GFP transgenic line labeling i-cell stem cells used for this analysis. **b** FACS-sorting of the i-cell population and non-i-cell population. **c** Size distribution of genomic insertions. **d** Number of cell type specific and shared insertions. Some of the insertions were further annotated as TEs highlighted in red. **e** Major types of TEs contributing to the genomic insertions. **f** TE families which comprise the genomic insertions of the i-cell population. The core 14 TE families are shown in the gray circle. **g** Counts of TE subfamilies in the 494 TE insertions (x-axis) and 275 TE insertions (y-axis). **h** Expression profile of TEs showing genomic insertions in the stem-cell lineage, based on reanalysis of publicly available scRNA-seq [54]. **i** Distribution of TE genomic insertions in intergenic regions, introns, and exons among cell populations. **j** Proportion of TE genomic insertions overlapping with open chromatin regions (OCRs). The bar plot shows the percentage of TE insertions that fall into OCRs, highlighting their potential regulatory significance. **k** Relationship between TE genomic insertions and expression variance. The y-axis represents the standardized expression variance of genes with or without nearby A-TE or non-A-TE genomic insertions in the present whole-genome sequencing of stem cells. The standardized expression variance of genes was calculated from the publicly available scRNA-seq data [54]. **l** GO-term enrichment analysis of closest genes to the insertions detected in the i-cell as well as non-i-cell (ecto/endo stem cells) lineage genomes

*Hydra*, primarily reproducing asexually through budding, can also resort to sexual reproduction. In laboratory conditions, transgenic hydra strains are propagated asexually, with offspring inheriting genomes from three parental stem cell types. Over time, these strains may accumulate unique transposon insertions in each stem cell type’s genome (Additional file 1: Fig. S7a). Utilizing this aspect of hydra biology, we investigated the genomic diversity caused by TE insertions in stem cells using the Cnnos1::GFP hydra transgenic line, which labels interstitial stem cells under the Nanos promoter (Fig. 4a) [53, 72]. A clonal population propagated through budding from a single founding polyp for more than 10 years was subjected to FACS to isolate GFP-positive (i-cells) and GFP-negative cells (non-i-cells), followed by Nanopore PromethION long-read sequencing for each group, yielding 15.1 Gb and 16.3 Gb of long reads for i-cells and non-i-cells, respectively (Fig. 4b, Additional file 1: Fig. S7b). These reads were aligned to the AEP genome sequence for variant calling with filtering (Additional file 1: Fig. S7b, Methods).

We identified 54,857 genomic insertions across both populations, with 6506 larger than 1 kb, indicative of active TEs (Fig. 4c, Additional file 2: Table S8). Notably, 726 insertions were unique to i-cells, 423 to non-i-cells, and 5357 were shared (Fig. 4d). The higher number of TE insertions in the i-cells correlates with the faster propagation rate of i-cells in homeostatic tissue [73, 74]. TEs constituted a major part (69.7%) of these insertions, with notable contributions from the A-TEs (Fig. 4e, f). Among the TE insertions, 68.0% (494 insertions) were in the i-cell population, 65.0% (275 insertions) in the non-i-cell population, and 70.3% (3767 insertions) shared by both populations, respectively (Fig. 4d). The 5357 insertions including the 3767 TE insertions which were observed both in i-cell population and non-i-cell population can be regarded as reflecting the genetic background difference between the transgenic line and the AEP line used for the reference genome (Fig. 4d). Among these TE insertions, DNA elements were the most numerous, followed by LINE elements, and then LTR elements (Fig. 4e). These TE insertions were composed of 55 TE families, 50 TE families, and 81 TE families for the i-cell population, non-i-cell population, and shared population, respectively (Fig. 4d). All of the populations included the A-TEs (Fig. 4f–h, Additional file 1: Fig. S7c, Additional file 1: Supplementary Note 2).

Using single-cell transcriptome and open chromatin data, we investigated the role of TE insertions in gene expression variation relative to insertion regions. We found that insertions generally happen more frequently in the intergenic regions for both i-cell and non-i-cell lineages, compared to the genomic average of fixed TE copies (Fig. 4i). However, striking differences were found between TE insertions in their open chromatin insertion preferences depending on stem cell lineage (Fig. 4j). TE insertions in the i-cell lineage occurred less frequently in open chromatin regions, with only 2.4% overlapping ATAC-seq peaks (*p* value = 0.002, Fisher’s exact test; Fig. 4j). In contrast, the non-i-cell lineage exhibited a higher frequency of TE insertions overlapping with ATAC-seq peaks compared to other lineages (over 5% of TE insertions, Fig. 4j). Corroborating this finding, A-TEs contributed to less gene expression variation in the i-cells compared to the other lineages (Fig. 4k). Our data suggest that open-chromatin TE insertions tend to be associated with higher expression variation in the somatic lineages. Furthermore, this suggests that TE insertions are under tighter regulatory control in the i-cells. The activity of TEs in hydra stem cells is known to be regulated by the PIWI-piRNA pathway [75, 76]. Consistent with this, scRNA-seq data shows that Piwi2 is expressed in three types of stem cells, with particularly high expression in i-cells (Additional file 1: Fig. S7d).

These data are supported by distinct categories of genes affected by stem-cell lineage-specific insertions. Analysis of the genes near the TE insertion loci specific to the i-cell population revealed 20 statistically significantly enriched gene ontology (GO) terms such as cell proliferation (left panel in Fig. 4l). This enrichment was highlighted by the i-cell specific A-TE hAT-Tip100 insertion in the intron of 3-phosphoinositide-dependent protein kinase 1 (PDPK1) [77, 78] (Fig. 4l, Additional file 1: Fig. S7e). This hAT-Tip100 genomic insertion contains a target site duplication, suggesting that this insertion occurred relatively recently (Additional file 1: Fig. S7f). Similarly, 22 gene groups associated with TE insertions specific to non-i-cells were identified, with GO terms related to substance transport such as protein transport and peptide transport being significantly enriched (right panel in Fig. 4l).

Similarly, we performed stem-cell lineage-specific whole-genome sequencing on the ecto-GFP/endo-RFP line [79], which expresses GFP in ectodermal epithelial stem cell lineage and RFP in endodermal epithelial stem cell lineage and has been propagated by budding since 2011 (Additional file 1: Fig. S8a–d, Additional file 1: Supplementary Note 2). We identified 409 TE insertions (54 families), 523 TE insertions (61 families), and 464 TE insertions (59 families) specific to the ectodermal stem cell lineage cell population, endodermal stem cell lineage cell population, and non-epithelial cell population, respectively (Additional file 1: Fig. S8c, d, Additional file 2: Table S9). Similar to the TE insertion patterns in the Cnnos1::GFP line (Fig. 4f, g, Additional file 1: Fig. S7c), these insertions were predominantly from the A-TEs (Additional file 1: Fig. S8e–h). However, they were associated with genes belonging to different GO categories, such as cilium organization in ectoderm, chromosome organization in the endoderm, and monoatomic ion transport in the non-epithelial cells (Additional file 1: Fig. S8i), suggesting different targeting of A-TE in distinct stem cell lineage genomes.

In summary, our lineage-specific whole-genome sequencing of stem cells has unveiled loci for TE insertions associated with distinct gene ontologies and predominantly influenced by the A-TEs. In addition, the contribution of the A-TEs to the general transgenic line differences suggests their accumulation in the germline.

### Deep evolutionary conservation of the A-TE families

**Fig. 5:**
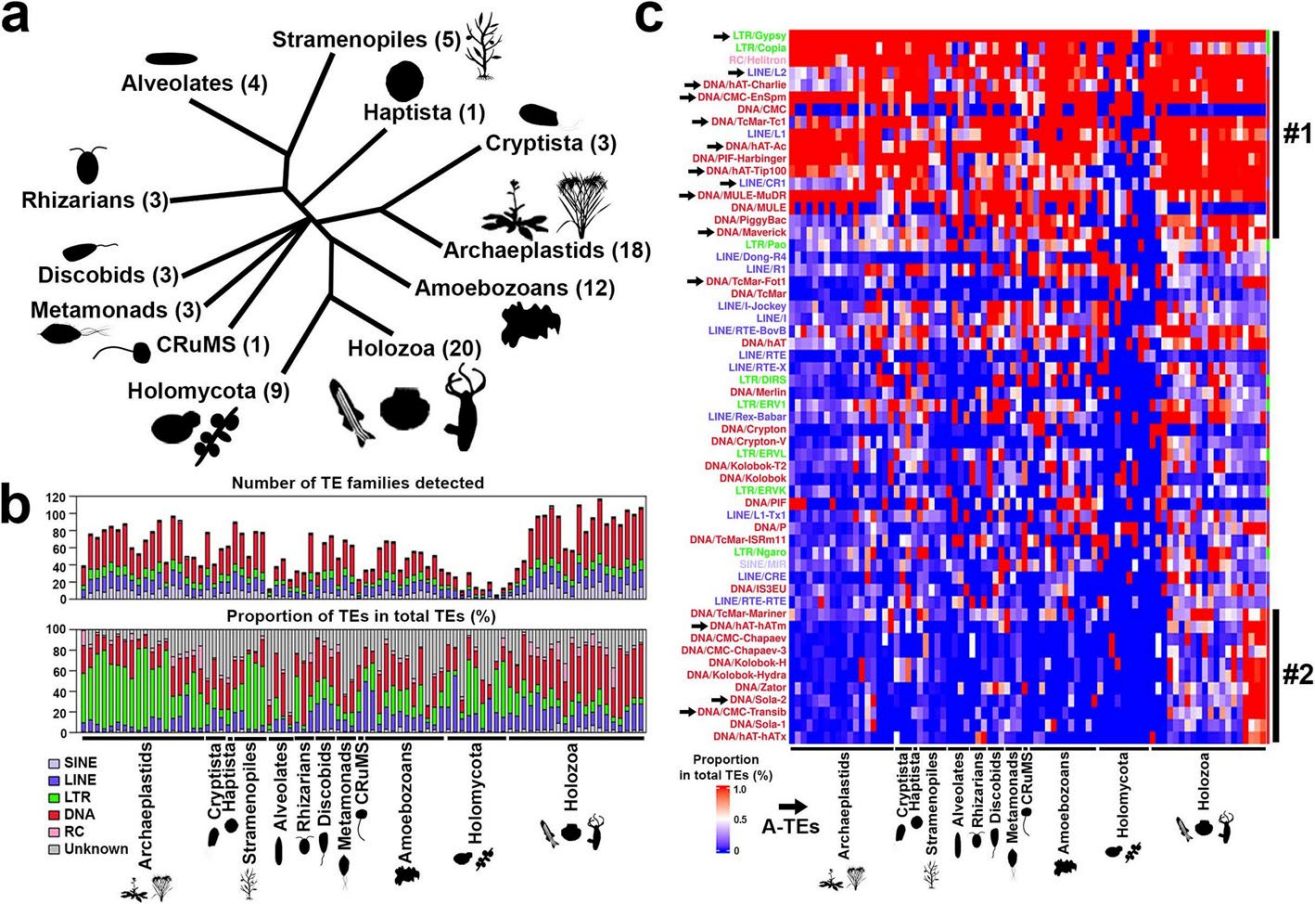
Deep TE family homology and retention of genomically active TEs across eukaryotes. **a** The cladogram of the species used in this analysis. The topology of the cladogram is derived from previous research [80, 81]. **b** Number of TE families detected in each species (upper panel) and proportion of TEs in total TEs in each species (lower panel). **c** The heatmap shows the extent to which the genome coverage of each TE family constitutes the overall genome coverage of TEs in each species. TEs belonging to cluster #3 in Additional file 1: Fig. S10 are shown. The cluster #1 (eukaryotic core TEs) and #2 (active in *Hydra*) correspond to those in Additional file 1: Fig. S10. The 14 TEs of the A-TEs are indicated by arrows

We investigated whether A-TEs are present across a wide clade in the genomes of animals and their outgroups to test the phylogenetic scope of their putative activity beyond hydra. For this purpose, we developed a custom deep homology search, incorporating hidden Markov model-based sequence searches to generate a custom repeat library with TE annotation (Additional file 1: Fig. S9a, Methods). Our pipeline has substantially improved the detection of TEs in the genomes of three unicellular holozoans (*Creolimax fragrantissima*, *Capsaspora owczarzaki*, *Salpingoeca rosetta*) compared to the standard methodology (Additional file 1: Fig. S9b–d). We analyzed TE homology and retention in 82 eukaryotic species representing all major eukaryote clades [80, 81] (Fig. 5a and Additional file 1: Fig. S10, Additional file 2: Tables S10, S11). A total of 178 TE families were detected in at least one species analyzed (Fig. 5b and Additional file 1: Fig. S10). We discovered a cluster of several TE families with overrepresentation across all the species (cluster #1 in Fig. 5c and Additional file 1: Fig. S10). We found that at least 58.8% (ten out of 17 TE families) of these families are members of A-TEs (arrows in cluster #1 in Fig. 5c and Additional file 1: Fig. S10). Only species with very compact genomes lacked a clear A-TE signature (Fig. 5c and Additional file 1: Fig. S10). The pan-eukaryotic distribution of these TE families suggests a very ancient conservation and roles of these A-TEs in shaping chromosomes that might predate the current metazoan-unicellular homologies [2]. We term these families core eukaryotic TEs. We found that the other part of A-TEs are located within one of several pan-metazoan clusters (cluster #2 in Fig. 5c and Additional file 1: Fig. S10). While this cluster has a particularly high activity in the genus *Hydra*, these TE families are also abundant in other metazoan species and may thus represent an active core metazoan TE set.

We found evidence that core TEs are continuously shaping animal chromosomes. We profiled the reported genome expansion in the zebrafish genome [82] and found that the core eukaryotic TE cluster members are heavily involved in the *Danio rerio* genome expansion contributing to 30.0% of the genome (Additional file 2: Table S11). We found that the core metazoan TE set is present but not as prominently active in the zebrafish genome as in the brown hydra genomes (cluster #2 in Fig. 5c and Additional file 1: Fig. S10). Similarly, nine out of 17 core eukaryotic TEs have contributed to the ancient coleoid cephalopod genome expansion and evolution [83]. However, we also found other TE clusters that are expanded in species-specific manners, that are not covered by A-TEs and may thus constitute divergent lineage-specific active TE core sets that were not captured in our hydra-centric analysis, including several ERVs and L1 families (Fig. 5c and Additional file 1: Fig. S10, Additional file 2: Tables S12–S15, Additional file 1: Supplementary Note 2).

Taken together, our data suggest that A-TEs are forming evolutionary conserved core sets of self-propagating elements on ancient chromosomes of eukaryotes and metazoans. The same core TE set might be employed multiple times in evolution contributing to independent genome expansions. Beyond the highly conserved eukaryotic core set, our data also suggest the presence of several other metazoan core TE sets, including one that contains the remainder of hydra A-TEs.

### Core TEs as slow-paced genomic expansion drivers

The distinction in abundance and repeat class composition of A-TE and non-A-TEs in animal and eukaryotic genomes point to two distinct regimes at which these elements can propagate. First, among the 82 investigated animal genomes, including those with the previously well described genome expansions, the variation of retroelement (LTR, LINE, SINE) contribution to genome sizes is most striking (Additional file 1: Fig. S10). Animals with “giant genomes” that have been sequenced so far show remarkable genome expansions by retroelements via copy-and-paste mechanisms [8, 9, 42, 84]. For example, axolotl (*Ambystoma mexicanum*) has a massive genome of 32 Gb, of which 65.6% consists of repetitive sequences, with LTRs accounting for 59% of the annotated repetitive elements [8]. The Australian lungfish (*Neoceratodus forsteri*) has a genome size of 37 Gb, with 90% being repetitive elements, and LINEs (mostly CR1 and L2 elements) dominating 25.7% of the genome [9]. Fixation of so many insertions, often contributing to substantial chromosome size increases, likely happens during periods of relaxed selection pressure, such as small population sizes or bottlenecks [85]. Conversely, our data demonstrate the presence of a much slower, but consistent chromosome expansion trend that is accomplished by the few A-TE families. Using comparison of the AEP and 105 strains, we show that these A-TEs can also propagate under normal sexual reproduction conditions. The functional role of A-TEs appears to be different depending on the stem cell lineage (Fig. 4, Additional file 1: Fig. S8). However, their effect may accumulate over macro-evolutionary timescales. In our investigation of chromosomal conformation capture, we observed that bins with higher A-TE coverage exhibited stronger chromatin interaction signals compared to those with lower A-TE coverage (Additional file 1: Fig. S11). Bins with higher A-TE coverage furthermore had significantly higher interactive ranges compared to other A-TE depleted bins (1.3 Mb vs 0.7 Mb, *p* value < 2.2e − 16, Wilcoxon rank sum test, Additional file 1: Fig. S11e). This may suggest that A-TEs could have much broader and subtle functions than individual gene expression control, being involved in overall chromatin organization, such as tethering elements through long-distance chromatin interactions [86] or other conformational states [87].

The role of A-TEs in the general chromosomal organization may also be supported by the observation that most, if not all, of the A-TE families are vertically transmitted and have been retained independently in multiple animal genomes. This is supported by the total number of repeat copies present across many metazoan genomes that can be traced to these families (Fig. 5c and Additional file 1: Fig. S10). Additionally, repetitive elements that have been reported to be horizontally transferred in hydra (Hvmar1 and Hvmar2) [88] do not map to any of the A-TE families. A-TEs also show significantly greater sequence divergence compared to non-A-TEs (*p* value < 2.2e − 16, Wilcoxon rank sum test) and nested insertion patterns (Additional file 1: Fig. S12) [89], indicative of more ancient insertions. While we cannot rule out that some of the younger A-TE copies originated via horizontal transfer, our analyses indicate a general tendency of vertical transmission and retention of these families.

## Discussion

How TEs have shaped and continue to affect genomes of animals remains an understudied question due to the large variance in genomic representation of these elements, and, more crucially, the lack of appropriate model systems to profile their activity and function. To address this question from both functional and evolutionary perspective, we utilized the cnidarian hydra with its distinct stem cell lineages that yield, under asexual reproduction, three independently evolving genomes within the same individual. With transgenic access to these genomes and cell type transcriptomics, we were able to reveal the putatively active TEs (A-TEs), the key TE components of these genomes and cell types, and to assess their expression and genomic insertion dynamics.

In this study, we provide direct evidence that the stem cells of hydra have diversified genomes due to specific sets of full-length and transcriptionally active TEs (A-TEs). Over a dozen of such TE families are actively and distinctively shaping genomic landscapes in interstitial, ectodermal, and endodermal stem cell lineages. Through insertion into sites in and around genes involved in various cellular functions, these insertions are likely to contribute to generating variation in expression of these genes. For example, the observation of TE insertions near genes involved in cell proliferation may suggest a race between TEs to make their host cell outcompete others. This mechanism may be of crucial selective advantage for an asexually reproducing animal such as hydra.

Leveraging the key phylogenetic position of hydra and using a broad eukaryotic species sampling, we also reveal that A-TEs are evolutionarily highly conserved either at the eukaryotic or metazoan levels, making them “core” evolutionarily preserved and active TEs of these genomes. This finding is striking, as so far deep homology of TEs has been mainly investigated at the superfamily level, but homology at family level has been largely inaccessible due to species sampling and the lack of high-quality genomic resources. Our findings suggest that only a few dozen TE families show ancient conservation and consistent genomic content across many deeply branching taxa. Other TE families either play a much smaller role in shaping genomes across larger phylogenetic scales or contribute to substantial genomic expansions in particular lineages. Our data suggest that genome expansions can be seen as a cumulative act of these distinct TE expansion regimes, generating a plethora of novel genomic material and driving chromosomal expansions. We also showed evidence that the A-TE aided expansion regime is more involved in long-range chromosomal contacts and thus may contribute to the overall chromosomal compaction.

At the metazoan level, beyond the core set dominated by the A-TEs identified in this study, the evolutionary comparison also hints at the existence of a few other core metazoan sets. We suggest that similar core sets from this widely conserved metazoan pool of TEs may exist and be employed in independent species-specific slow genome expansions. This suggests the possibility that animal chromosomes evolve through activation waves of particular core TE sets, while keeping the ancient chromosomal homologies stable. While the mechanistic insights into the evolutionary genome dynamics remains to be fully elucidated, the evolutionary conserved PIWI-piRNA pathway-mediated TE repression might be coupled with it [76]. Together, these findings shed new light on old questions on the vast TE diversity, modalities of metazoan genome evolution, and test how activations of core TE sets are contributing to divergent macro-evolutionary trajectories of animal genomes [90].

## Conclusions

In this study, we investigated the activation and insertion dynamics of TEs across different stem cell lineages of hydra, a basally branching metazoan providing for a crucial comparative point to other animal genomes. We found that A-TEs, primarily composed of DNA elements, explain the minor genome size differences between different hydra strains and show distinct insertion profiles across the three hydra stem cell genomes. The majority of these TE families are evolutionarily conserved and are highly represented across most animal genomes. While these families are not the main contributors to major animal genome expansions, which are mainly driven by retroelements, we show evidence that insertions of A-TEs can impact gene expression, and that their accumulation over longer evolutionary timescales is correlated with stronger distal genomic contacts. This study provides evidence for a core set of TE families that has consistently accompanied animal chromosome evolution, opening up avenues for their further functional dissection.

## Methods

### Animal husbandry

Hydra strains were kept in individual containers filled with hydra medium at 16 °C. Hydras were maintained under a continuous cycle of 14 h of light and 10 h of darkness. Hydras received regular feedings of newly hatched brine shrimp. The hydra medium was changed daily. The Cnnos1::GFP strain and the ecto-GFP/endo-RFP strain were from previous studies [72, 79].

### Generation of the AEP × 105 strain

Males of the AEP Hym176B promoter::GFP line from Toshitaka Fujisawa were crossed with NE07a females. One of the progeny from this cross carried the transgene; this animal was used to establish a line. This line was heterozygous for the transgene. Females from this line were crossed with males from the Landing Site 6.1 line, which is an AEP transgenic line homozygous for the actin promoter::DsRed2 transgene and is six generations inbred. From this cross, lines were established that carried both transgenes (heterozygous for GFP and heterozygous for DsRed2). Males from these lines were crossed to females from the SS1 AEP line (an AEP F1 line that is “supersexy”). Subsequent studies using progeny from these crosses showed that the transgenes were on the same chromosome and that in one of these progenies a crossover had occurred that linked the two transgenes on that chromosome. This line was 7/8 AEP and 1/8 NE07a, and it was heterozygous for the chromosome containing the linked transgenes. Males from this line were crossed with 105 females to generate the AEP × 105 hybrid strain. The AEP × 105 strain that was obtained did not carry the transgenes.

### Nanopore sequencing for genome assembly

Extraction of high molecular weight DNA from a snap-frozen sample of hydra polyps of the AEP × 105 strain and Nanopore sequencing on an Oxford Nanopore Technologies PromethION were carried out by the DNA Technologies and Expression Analysis Cores at the UC Davis Genome Center, which is supported by NIH Shared Instrumentation Grant 1S10OD010786-01. Following the initial run, the flow cell was given a nuclease flush and reloaded for a second round of data collection. The two datasets were combined for subsequent use.

### Omni-C sequencing

A snap-frozen sample of hydra polyps of the AEP × 105 strain was provided to Cantata Bio (Scotts Valley, CA) who produced and sequenced an Omni-C library.

### Iso-Seq sequencing

RNA was extracted from hydra polyps of the AEP × 105 strain using the method of Chomczynski and Sacchi [91]. Following resuspension in DEPC-treated water, the RNA was precipitated with 2.5 M LiCl overnight at 4 °C. The RNA was pelleted, washed twice with 70% ethanol, resuspended in DEPC-treated water, and stored at − 80 °C. Synthesis of cDNA, preparation of the SMRTbell template, and sequencing (two SMRT cells) were done at the Genomics Research and Technology Hub (GRT Hub) at UC Irvine on a Pacific Bioscience Sequel II sequencer.

### Generation of haplotype-resolved genome assembly of the AEP × 105 F1 hybrid

The heterozygosity of the AEP × 105 F1 hybrid genome was assessed from the Illumina short reads of the genome [1] using Jellyfish v2.3.1 [92], a k-mer counting software, with a k-mer size of 21. The k-mer spectrum was analyzed using GenomeScope [93]. The resulting k-mer profile displayed a single peak, suggesting that the AEP haplotype and the 105 haplotype were well diversified and resolving each haplotype genome is feasible (“pseudohomozygosity”). In the previous study, total 4420 contig sequences with the total size of 1.75 Gb were previously assembled from 23.6 Gb PacBio HiFi reads of the AEP × 105 genome [1]. The chromosome-level scaffolds were constructed with the combination of the 4420 contig sequences and the newly generated 26.2 Gb Omni-C (DNase I Hi-C) sequencing reads. The read quality of the Hi-C reads was evaluated using FastQC v0.12.1. The Hi-C reads were mapped to the contig sequences using Juicer v1.6 [94] with default parameters. Subsequently, Hi-C scaffolding was performed using the 3D-DNA pipeline v180922 [95] with parameters the default parameters followed by curation of resulting scaffolds using Juicebox Assembly Tools v1.11.08 [96]. This Hi-C scaffolding step produced 30 chromosomal-scale scaffolds which are corresponding to both of 15 haplotype chromosome sets. These scaffolds comprise 97.1% (1.70 Gb) of the initial contigs. By comparing with previously reported genome assemblies of the AEP strain [60] and the 105 strain [1], we confirmed that 15 of the scaffolds correspond to the paternal AEP haplotype genome, while the remaining 15 correspond to the maternal 105 haplotype genome. The AEP haplotype genome sequences had 1523 gaps, while the 105 haplotype genome sequences had 2300 gaps. Subsequently, all these gaps were filled with the newly generated 48 Gb of raw Oxford Nanopore reads. We identified telomere reads from the previous HiFi reads and newly generated Nanopore reads for this study and extended the terminal sequences of chromosomes. Resulting scaffolds were polished with previously obtained 35.6 Gb Illumina short reads of the AEP × 105 F1 hybrid [1]. In the final genome assembly, each haplotype was gapless and featured telomere-subtelomere sequences at both ends of all 15 chromosomes. The AEP haplotype genome was 912 Mb in size, while the 105 haplotype genome was 834 Mb in size. Compared to previously reported data [1, 60], the AEP haplotype exhibited an increase of 10.9 Mb, while the 105 haplotype showed an increase of 16.2 Mb. Completeness of the genome assemblies examined using BUSCO v5.4.6 and the metazoa_odb10 datasets [97]. The BUSCO scores on genome sequences for both haplotype genomes were 91.7%, an improvement over the previously reported AEP genome assembly and 105 genome assembly scores of 91.5% and 91.4% [1, 60]. Based on these findings, the genome assembly appeared closer to a complete genome assembly. We have named this genome assembly HydraT2T. The sequences of the AEP haplotype genome and the 105 haplotype genome have been deposited in the NCBI Genome database under the accession numbers GCF_038396675.1 (HydraT2T_AEP) and GCF_037890685.1 (HydraT2T_105).

### Generation of a custom repetitive element library

Since the majority of TEs are inactivated and degenerated due to accumulation of mutations such as base substitutions, insertions, and deletions, TE annotation in a pan-metazoan scale is challenging [98, 99]. At the first step of TE annotation, generating custom repeat libraries using RepeatModeler v2.0.5 [100] is a standard approach. A simple RepeatModeler run occasionally produces a library that contains lots of repetitive elements as “Unknown” elements. Analyzing the genome sequence with RepeatMasker v4.1.6 using this custom repeat library, which includes many “Unknown” sequences, inevitably results in a large number of repetitive elements being defined as “Unknown.” Since the process of RepeatModeler [100] generating consensus sequences for repetitive elements does not refer to the TE database such as Dfam [66], RepeatMasker using RepeatModeler custom library still detects the proper proportions of repetitive elements in the genome sequence. RepeatModeler classifies the consensus sequences of repetitive elements using its basic homology-based classification module called RepeatClassifier [100]. RepeatClassifier searches for homologous sequences within the Dfam sequences using the NCBI BLAST-based search (https://github.com/Dfam-consortium/RepeatModeler). If homologous sequences are not found in the database, RepeatMasker labels the remaining sequences as “Unknown” [100]. BLAST is a fast sequence search method that utilizes the dot matrix method. The hidden Markov model-based sequence search is another sequence search approach and can be generally applied for homology searches for evolutionarily distant sequences [101, 102]. The hidden Markov model can even be used to detect RNA viral genes with highly divergent sequences [103, 104]. Therefore, for the consensus sequences generated by RepeatModeler, we performed annotation using the nhmmscan v3.4 [101], which uses a more sensitive hidden Markov model instead of BLAST, which RepeatClassifier uses. We searched for the best hit in the Dfam 3.8 database [66] for each consensus sequence of repetitive elements. For each consensus sequence of repetitive elements, the top hit from the nhmmscan search against Dfam was used as the TE annotation. Consensus sequences that did not have a hit identified in Dfam during the sequence search were labeled as “Unknown,” similar to how the RepeatClassifier operates. We have additionally validated our repeat library annotation using other external sources, including Repbase [67] and Gypsy Database [68].

### TE detection in the genomes

With the custom repetitive element library, RepeatMasker [105] was performed on the genome sequences with the parameters of -parallel 70 -gff -a -lib “repetitive element library file” -dir -xsmall. For the benchmark analysis, we also generated another repetitive element library using RepeatModeler with default parameters. We collected chromosome-level genomic scaffolds of three unicellular holozoans, including *Creolimax fragrantissima*, *Capsaspora owczarzaki*, and *Salpingoeca rosetta* from the supplemental datasets in the previous study [2] (10.5061/dryad.dncjsxm47). Then, we performed genome masking using RepeatMasker with two different repetitive element libraries: the custom repetitive library and the default repetitive library. The output files from RepeatMasker were processed using custom scripts, which computed various statistics, including TE coverage across the genome.

### Analysis of nested insertion patterns for A-TEs

For the analysis of nested insertion patterns of A-TEs, the TinT tool [89] was used. The distribution of each A-TE was extracted from the RepeatMasker output and used as input for the TinT tool with default parameters to infer nested insertion relationships.

### Genome annotation

To predict protein-coding genes in the genome assembly, we aligned Iso-Seq reads to the genome assemblies using the minimap2 v2.26-r1175 [106] with options -ax splice -uf -C5. We then performed genome annotation using BRAKER2 v 2.1.6 [107] on the repeat-masked genome assemblies masked by RepeatModeler [100] and RepeatMasker [105]. The UTR sequences were further added to the gene prediction with the parameters of –addUTR = on –skipAllTraining.

### Synteny analysis

Reciprocal blast best hits were used to define orthologs between species. The distribution of orthologs across species was plotted using the R package macrosyntR v0.3.3 with default parameters [108]. Collinear synteny blocks were identified using MCScanX with default parameters [109]. Collinear synteny blocks were visualized using SynVisio [110].

### Stem-cell lineage-specific whole-genome sequencing

One hundred hydra individuals with clonal propagation were carefully collected and transferred to a 2.0-ml tube containing dissociation medium [111]. The hydra were then gently dissociated by incubating them in Pronase solution (0.1% Pronase in hydra medium) for 90 min at 18 °C. Throughout the incubation phase, gentle agitation and pipetting were performed to facilitate the disassociation procedure. After the incubation, the Pronase activity was neutralized by adding an equal volume of BSA-containing hydra medium. The dissociated cells were subsequently washed three times with 1 ml dissociation medium. Finally, the dissociated cells were collected by centrifugation at 2000 rcf for 3 min at 4 °C and then resuspended in 1 ml dissociation medium. FACS were performed with BD FACSMelody™ Cell Sorter at the Max Perutz Labs BioOptics FACS Facility. The FACS-sorted cells were suspended in a lysis buffer (pH 8.0) containing 10 mM Tris, 100 mM EDTA, and 0.5% SDS to lysis the cell and solubilize genomic DNA. Equal volume of phenol:chloroform:isoamyl alcohol (25:24:1, v/v/v) was added to the sample lysate, and the mixture was vortexed for 10 s to ensure proper emulsification. After mixing, the suspension was centrifuged at 12,000 rpm for 5 min at 4 °C to facilitate phase separation. The upper aqueous phase containing genomic DNA was carefully transferred to a new tube, avoiding the interphase. Equal volume of chloroform was added, and mixture was vortexed for 10 s. The suspension was centrifuged at 12,000 rpm for 5 min at 4 °C. The upper aqueous phase containing genomic DNA was carefully transferred to a new tube, and the same step was repeated once. The final aqueous phase was treated with equal volume of 2.5 volumes of room temperature isopropanol and 1/10 volume of 3 M sodium acetate (pH 5.5). The sample was mixed well. After incubation, the sample was centrifuged at 6000 rcf for 5 min at 4 °C. The supernatant was discarded, and the pellet was washed three times with 70% ethanol, air-dried, and finally resuspended in 100 μl TE buffer. The concentration and purity of the extracted genomic DNA were determined using a Nanodrop spectrophotometer. Library preparation and nanopore sequencing were performed at the Next Generation Sequencing core facility at the Vienna BioCenter.

### Nanopore read mapping to the genome sequence and variant calling for the stem-cell lineage whole-genome sequencings

Qualities of the Nanopore reads were evaluated using NanoPlot [112]. Nanopore reads were aligned to the hydra reference genome sequence [60] using minimap2 v2.26-r1175 with options of -ax map-ont –secondary = no [106]. Variant calling for each individual sample was performed using Sniffles v2.02 [113] with the –minsupport 2 option which produces a SNF file for each sample. Individual variant calls were merged using Sniffles v2.02 with options of –combine-low-confidence 0.01 –combine-low-confidence-abs 1 –combine-null-min-coverage 3 which produce a combined VCF file. The genomic insertions where all samples have genotypes were kept using bcftool v1.19 [114]. The insertions were further subset by bcftools v1.19 based on the combination of genotypes for each sample, for the downstream analyses. For insertion annotations, sequences of insertions longer than 1 kb were extracted and searched against the RepeatModeler repeat library using NCBI BLASTN [115]. The TE annotation of an insertion was determined based on the consensus sequence of the custom repeat library that formed an alignment of 500 bp or more with the TE sequence.

### Functional gene analysis

For functional gene analysis, protein sequences were retrieved from the genome sequence and the genome annotations using GffRead v0.12.7 [116]. The longest protein sequences were analyzed to identify homologous sequences within the NCBI-nr database, employing the NCBI BLASTN program with an *e*-value threshold of 1e − 05. The longest protein sequence for each gene was functionally annotated using eggNOG-Mapper v2.1.9 [117]. Genes proximal to TE insertion sites were identified using BEDTools v2.30.0 [118] with a subcommand of closest. We conducted Fisher’s exact test for the overrepresentation of GO terms within the group of genes proximal to TE insertion sites, utilizing the R library topGO v 2.54.0 [119]. Results of the statistical tests were plotted using the R library ggplot2 v 3.5.0 [120].

### RNA-seq analysis

To determine whether each Iso-Seq read is derived from TEs, each read sequence from Iso-Seq was searched against the repeat library generated by RepeatModeler [100] using NCBI BLASTN [115] with default parameters. Iso-Seq reads that formed alignments of over 1 kb with the sequences in the repeat library were called as TE-derived sequences. Furthermore, Iso-Seq reads defined as TE-derived were mapped to the genome sequence using Minimap2 v2.26-r1175 with parameters of -ax map-hifi –secondary = no, and the mapped regions were identified. Additionally, to reveal the transcription of TEs at a single-cell resolution, sequencing reads from a publicly available scRNA-seq dataset of hydra [54] were downloaded. From this dataset, only TE sequences belonging to the TE subfamilies (RepeatModeler consensus sequences) confirmed to be expressed by Iso-Seq and that were over 1 kb in length were used. With these TE sequences, the expression levels of TEs in each cell were quantified using Alevin v1.2.1 [121]. The resulting expression matrix was loaded using the CreateSeuratObject function of the R package Seurat v5 [122]. Cell type annotation was based on the previous reports [54, 60]. TEs showing cell type-specific expression were identified using the FindAllMarkers function of Seurat. Additionally, RNA-seq datasets for FACS-sorted cell populations [53] were mapped to the genome sequence using HISAT2 v 2.2.1 [123]. The read counts of each TE were evaluated with featureCounts [124], and transcripts per million were calculated for each TE. The expression of TEs showing cell type-specific expression obtained from scRNA-seq was checked. Heatmaps were plotted using the R package ComplexHeatmap v 2.18.0 [125].

### Identification of open chromatin regions

To identify open chromatin regions in the hydra genome, publicly available ATAC-seq reads (NCBI accession number: SRR18362011) from whole adult polyps were obtained from the NCBI SRA database. These reads were aligned to the hydra genome assembly using BWA-MEM [126]. Peak calling from the ATAC-seq read alignment was performed using MACS3 v 3.0.2 [127]. Overlapping TEs and ATAC-seq reads were analyzed using BEDTools v2.30.0 [118] with a subcommand of intersect.

### Analysis of chromatin organization

Omni-C reads were aligned to the hydra genome assembly, and chromatin contact intensities were quantified using the Juicer/3D-DNA pipeline [96]. HiC contact intensities were extracted with hic-straw v 1.3.1 [96] and then converted into a BED file. The overlap between HiC bins and TE loci was identified using BEDTools v2.30.0 [118] with a subcommand of intersect. Statistical tests and visualization of results were performed using R v4.3.2.

## Supplementary Information

Additional file 1: Figures S1–S12, Supplementary Notes 1–2.

Additional file 2: Tables S1–S15.

## Data Availability

The sequencing reads are available in the NCBI database under BioProject accession number PRJNA1085048. The genome assemblies for the AEP and 105 genomes are available in the NCBI database under BioProject accession numbers PRJNA1085304 and PRJNA1085315, respectively. The scripts for this manuscript are available at the Zenodo repository (10.5281/zenodo.15600058) [128]. The RepeatMasker output files generated in this study are publicly available on the Figshare repository (10.6084/m9.figshare.28775144.v2) [129]. The GenBank accession numbers of the publicly available ITS1-5.8S-ITS2 rDNA sequences used in this study are listed in Table S3 of Additional file 2. The NCBI genome accession number corresponding to the publicly available genome sequences analyzed in this study is listed in Table S10 of Additional file 2.
