# Supplementary material for: The dynamic genomes of Hydra and the anciently active repeat complement of animal chromosomes
Source: Genome Biol. 2025 Jul 1;26:186. doi: 10.1186/s13059-025-03653-z (PMC12220107; doi:10.1186/s13059-025-03653-z)
Supplement: Supplementary file 1 — Additional file 1: Figures S1–S12, Supplementary Notes 1–2. [file 13059_2025_3653_MOESM1_ESM.pdf]

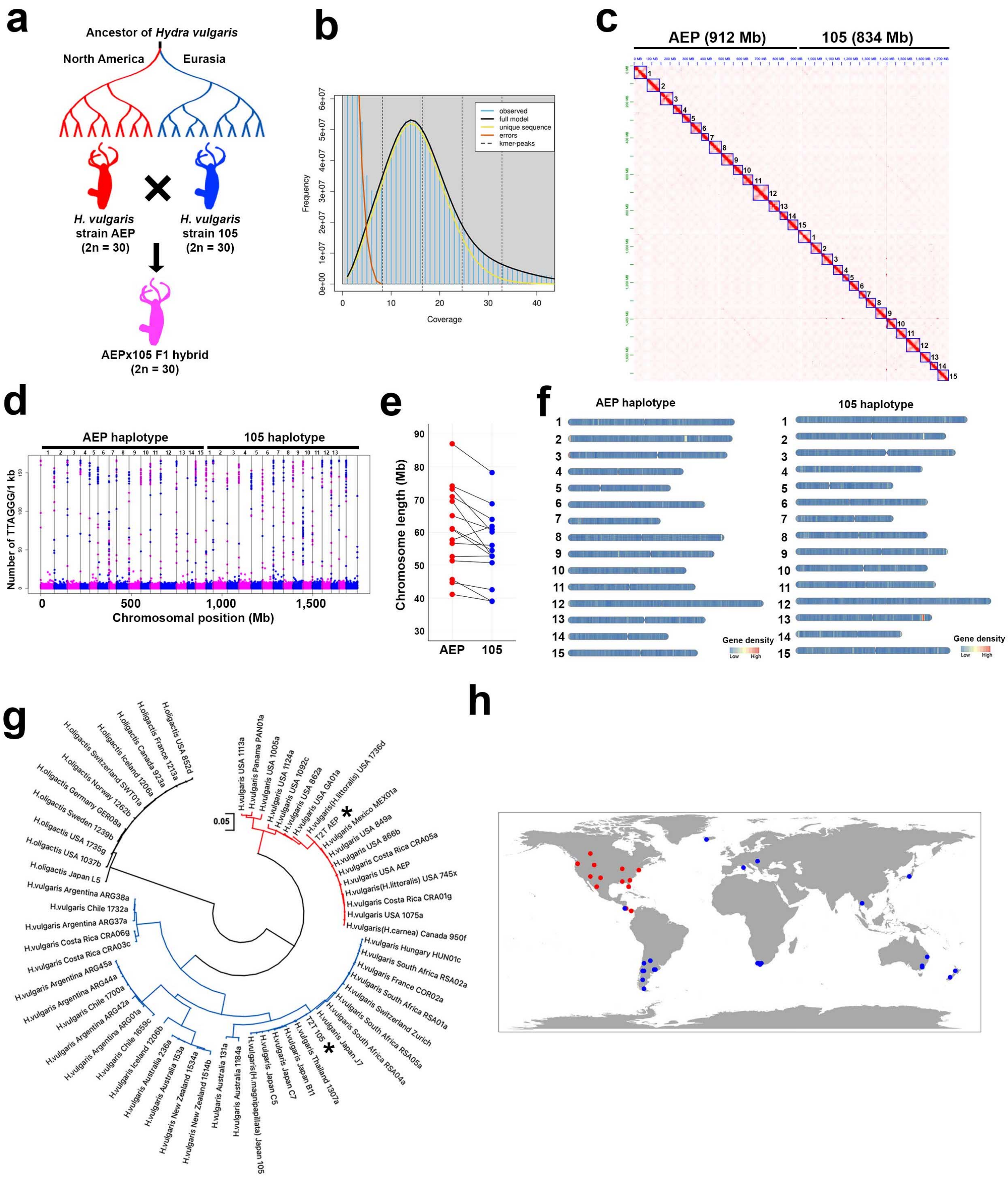

Fig. S1. The 30 chromosomes of the AEPx105 F1 hybrid

(a) Generation of AEPx105 F1 hybrid by crossing the AEP strain and the 105 strain. **(b)** The k-mer frequency distribution of the AEPx105 F1 hybrid genome. The k-mer (k = 21) occurrence was analyzed in the raw short-reads of the AEPx105 F1 hybrid genome[1]. **(c)** Hi-C contact map of the 30 chromosomes of the AEPx105 F1 hybrid. In each chromosome, interaction signals at both ends of the chromosome are observed, suggesting a Rabl-like configuration, an arrangement of

interphase chromosomes where centromeres and telomeres are located at opposing poles within the nucleus. **(d)** Distribution of telomeric repeats. The x-axis represents genomic positions and the y-axis shows the number of occurrences of telomeric repeats (TTAGGG) per kilobase pair. The plots of each chromosome are colored alternately to distinguish between the plots of the terminal regions of two different chromosomes. **(e)** Chromosome lengths of each haplotype genome. Plots between orthologous chromosomes are linked with black lines. **(f)** Chromosomes are colored according to gene density. The positions of the centromeres are indicated by notches. It is evident that each haplotype genome consists of metacentric or submetacentric chromosome. **(g)** Molecular phylogenetic positions of the AEP haplotype and the 105 haplotype. Maximum-likelihood tree based on sequences of the internal transcribed spacer (ITS) 1, the 5.8S ribosomal RNA gene, and ITS2 from the AEP haplotype, the 105 haplotype, and sequences from a public database. Samples of *Hydra oligactis* are colored black. Two major clades of *Hydra vulgaris* are colored red and blue. The ITS1-5.8S-ITS2 sequences of the AEP haplotype and 105 haplotype are indicated by asterisks. **(h)** Geographic distribution of the samples whose ITS1-5.8S-ITS2 sequences are publicly-available. Each dot represents individual sample, colored in accordance with the Maximum-likelihood tree.

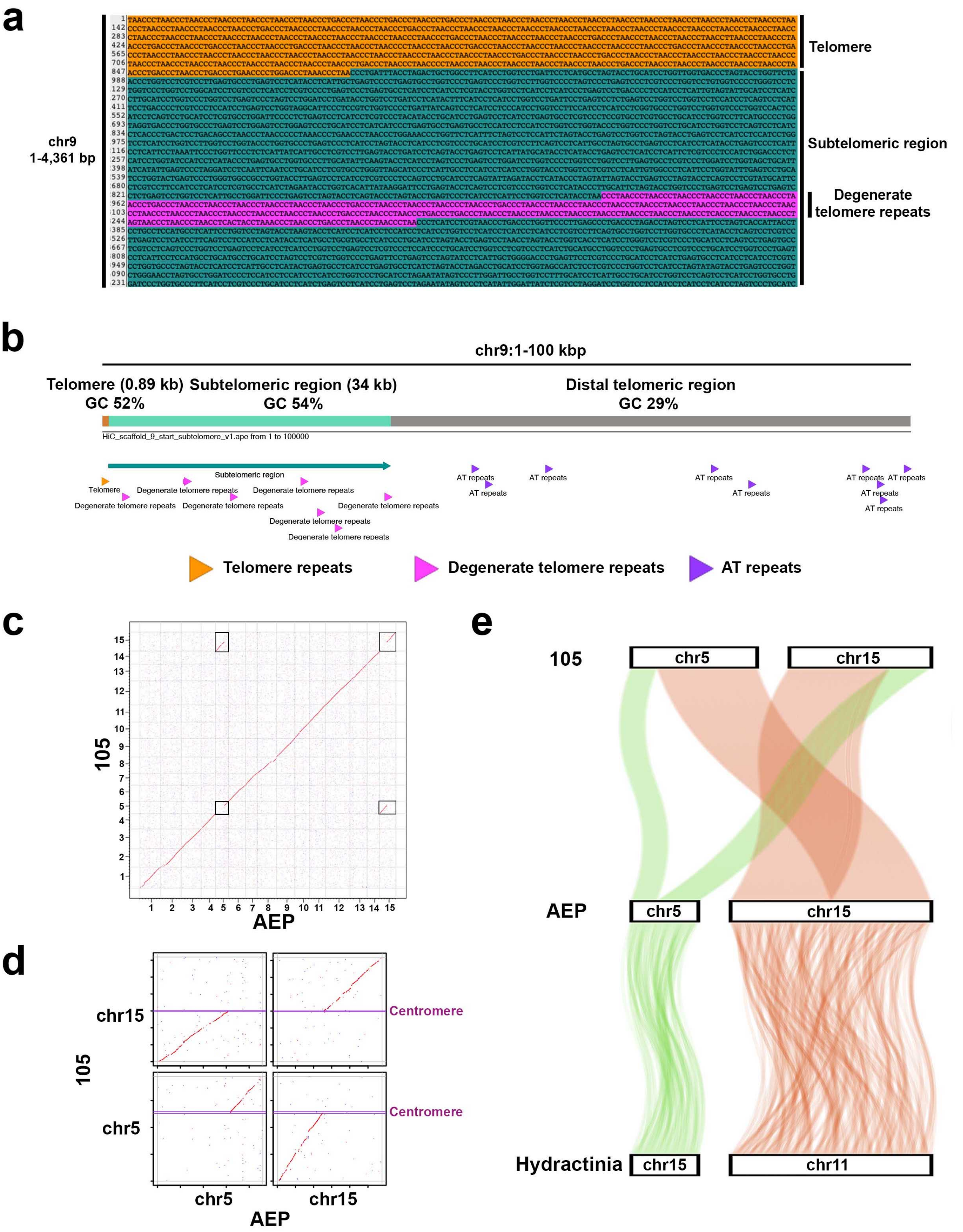
a
chr9
1-4,361 bp
Telomere
Subtelomeric region
Degenerate telomere repeats
b
chr9:1-100 kbp
Telomere (0.89 kb)
GC 52%
Subtelomeric region (34 kb)
GC 54%
Distal telomeric region
GC 29%
HiC_scaffold_9_start_subtelomere_v1.ape from 1 to 100000
Subtelomeric region
Telomere
Degenerate telomere repeats
AT repeats
Telomere repeats
Degenerate telomere repeats
AT repeats
c
105
AEP
d
chr15
chr5
105
Centromere
Centromere
chr5
chr15
AEP
e
105
chr5
chr15
AEP
chr5
chr15
Hydractinia
chr15
chr11

Fig. S2. The structure of hydra telomeric, subtelomeric and centromeric regions

(a) Telomere and subtelomere sequences in the 1-4,361 bp region of the chromosome 9. **(b)** Motif map of the telomere, subtelomere, and distal telomeric regions in chromosome 9. **(c)** Genome alignment between the AEP chromosomes and the 105 chromosomes. Alignments of the chromosomes involved in the translocation is highlighted with rectangles. **(d)** Genome alignment between chromosome 5 and chromosome 15 from the AEP haplotype genome and those from the 105 haplotype. The start and end of the centromeric regions are indicated by horizontal lines. **(e)** Highlight of the Robertsonian translocation. Each curved line indicates positions of orthologs.

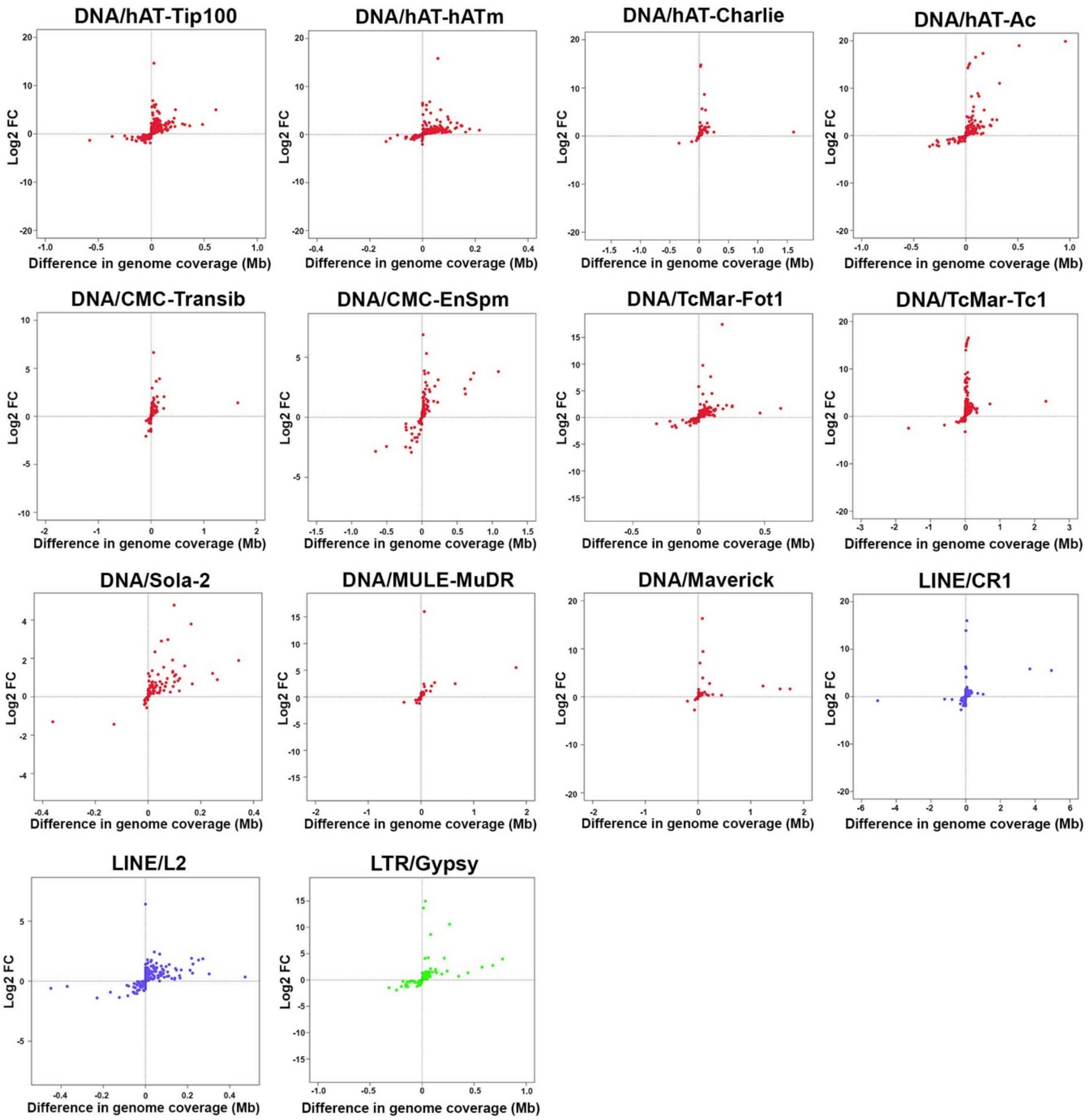

Fig. S3. Divergence of the genome coverage of subfamilies of A-TEs between the AEP and 105 genomes

The x-axis represents the difference in genome coverage of each TE subfamily between the AEP and 105 genomes (in Mb), while the y-axis displays the log2-transformed fold change (Log2 FC) in genome coverage of each TE subfamily between the two genomes. Each dot represents each subfamily.

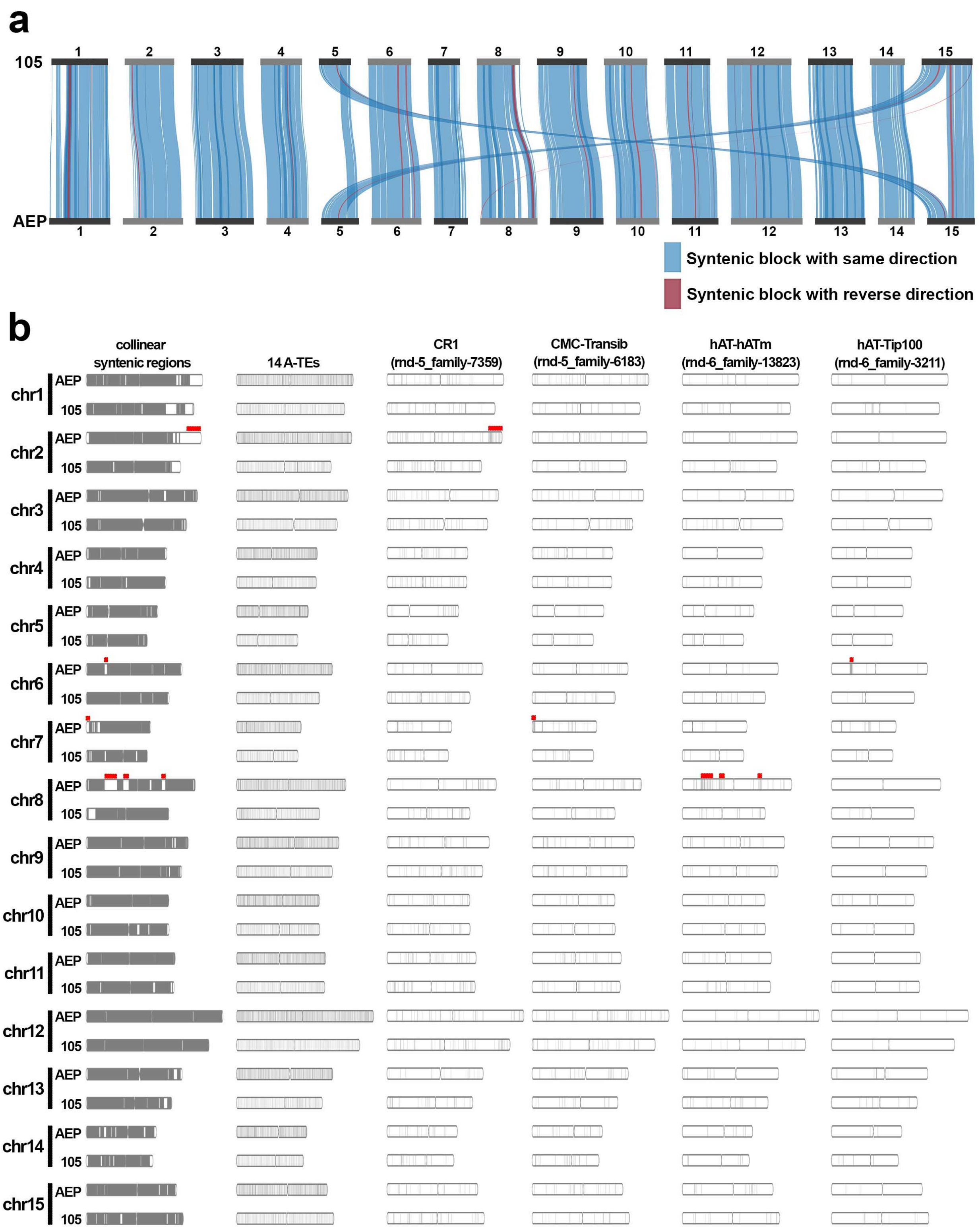

Fig. S4. Divergence of TE subfamilies between the AEP and 105 genomes

(a) Collinear syntenic blocks between the 105 chromosomes and the AEP chromosomes. Collinear syntenic blocks are shown as ribbon diagrams. Syntenic blocks with same chromosomal orientations are colored in blue. Syntenic blocks with reverse orientations are colored in red. The

length of the chromosomes is depicted as proportion to the assembly size of each chromosome. **(b)** Distributions of TE subfamilies across chromosomes. The leftmost panel shows the distribution of collinear syntenic regions in black. The second panel from the left represents the density plot for the 14 A-TEs. The remaining panels provide examples of the distributions of subfamilies of A-TEs. The red lines indicate genomic regions where collinear syntenic regions were not detected.

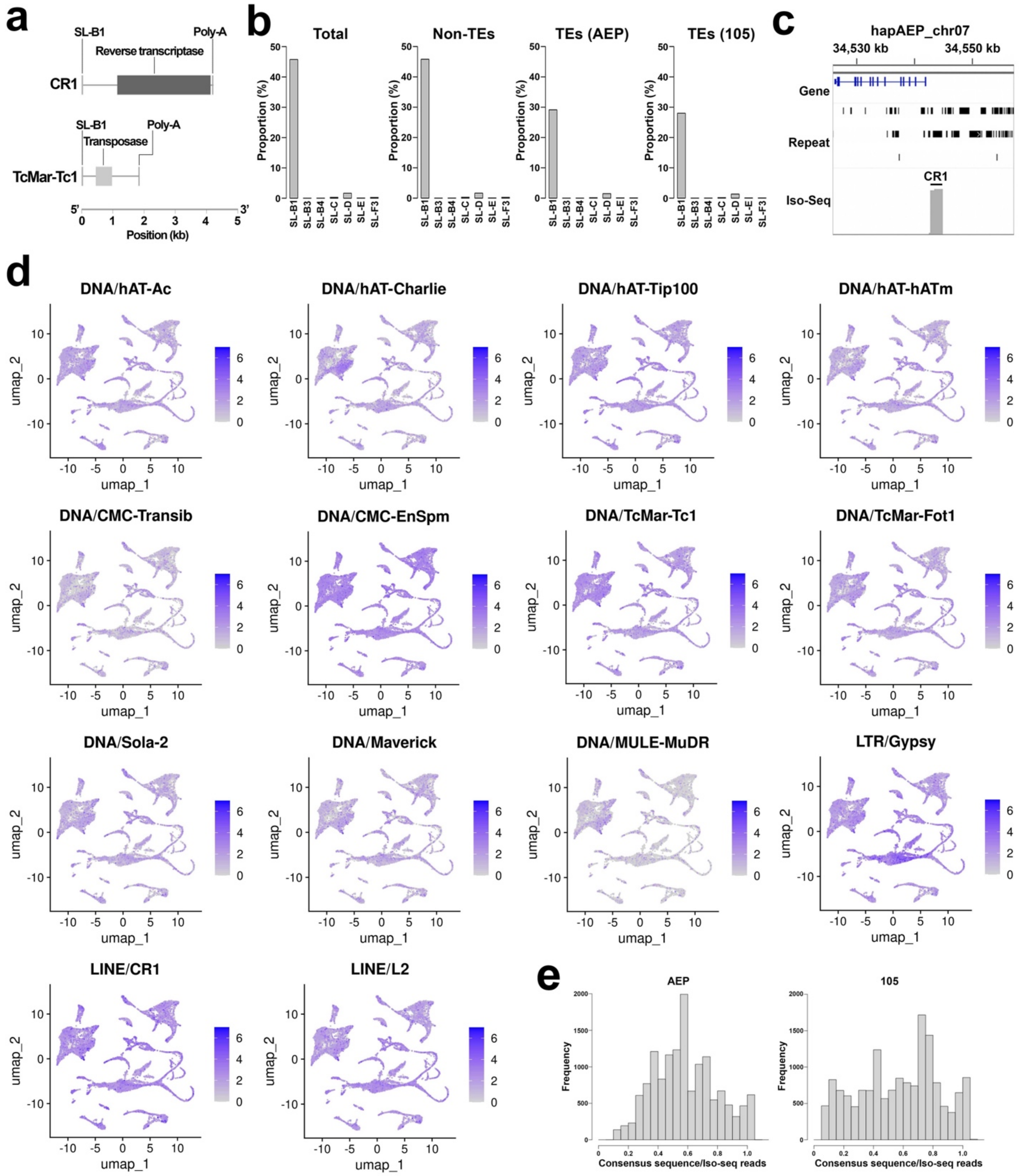

Fig. S5. Expression profiles of TE families

(a) Full length transcripts of the CR1 element and TcMar-Tc1 element with trans-spliced leader sequences (SL-B1[2]) attached at the 5' end**. (b)** Proportions of transcripts with trans-spliced leader sequences revealed by Iso-Seq. The x-axes represent types of trans-spliced leader sequences[2]. The y-axes represent the proportions of transcripts with trans-spliced leader sequences. **(c)** Representative locus with CR1 expression measured by Iso-Seq. **(d)** Expression profiles of the A-TEs at the family level. The color scale indicating gene expression levels is the same in all panels,

and the variations in color intensity are comparable across different panels. **(e)** Coverage of TE subfamily sequences in TE Iso-seq reads.

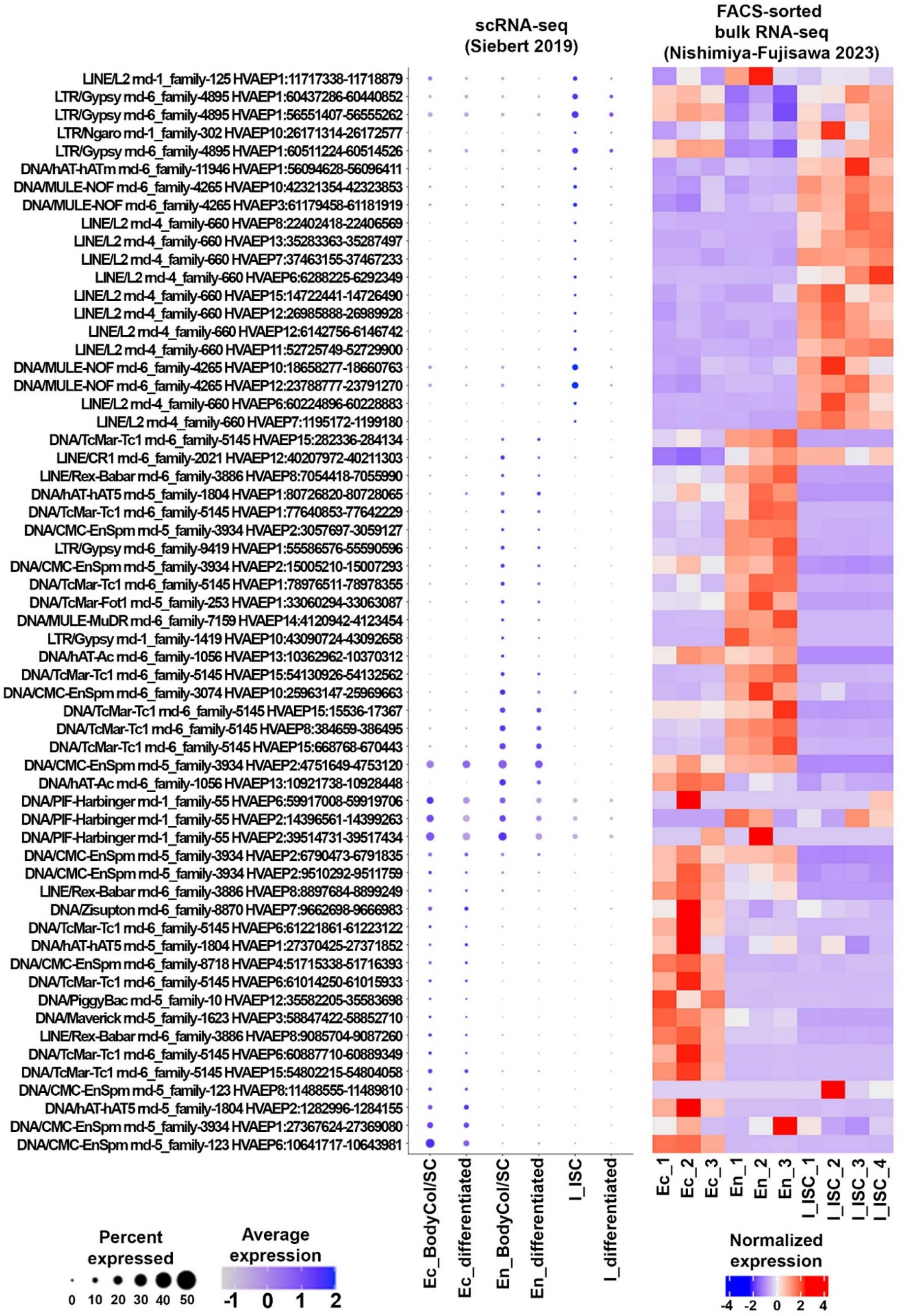
scRNA-seq
(Siebert 2019)
FACS-sorted
bulk RNA-seq
(Nishimiya-Fujisawa 2023)
LINE/L2 rnd-1_family-125 HVAEP1:11717338-11718879
LTR/Gypsy rnd-6_family-4895 HVAEP1:60437286-60440852
LTR/Gypsy rnd-6_family-4895 HVAEP1:56551407-56555262
LTR/Ngaro rnd-1_family-302 HVAEP10:26171314-26172577
LTR/Gypsy rnd-6_family-4895 HVAEP1:60511224-60514526
DNA/hAT-hATm rnd-6_family-11946 HVAEP1:56094628-56096411
DNA/MULE-NOF rnd-6_family-4265 HVAEP10:42321354-42323853
DNA/MULE-NOF rnd-6_family-4265 HVAEP3:61179458-61181919
LINE/L2 rnd-4_family-660 HVAEP8:22402418-22406569
LINE/L2 rnd-4_family-660 HVAEP13:35283363-35287497
LINE/L2 rnd-4_family-660 HVAEP7:37463155-37467233
LINE/L2 rnd-4_family-660 HVAEP6:6288225-6292349
LINE/L2 rnd-4_family-660 HVAEP15:14722441-14726490
LINE/L2 rnd-4_family-660 HVAEP12:26985888-26989928
LINE/L2 rnd-4_family-660 HVAEP12:6142756-6146742
LINE/L2 rnd-4_family-660 HVAEP11:52725749-52729900
DNA/MULE-NOF rnd-6_family-4265 HVAEP10:18658277-18660763
DNA/MULE-NOF rnd-6_family-4265 HVAEP12:23788777-23791270
LINE/L2 rnd-4_family-660 HVAEP6:60224896-60228883
LINE/L2 rnd-4_family-660 HVAEP7:1195172-1199180
DNA/TcMar-Tc1 rnd-6_family-5145 HVAEP15:282336-284134
LINE/CR1 rnd-6_family-2021 HVAEP12:40207972-40211303
LINE/Rex-Babar rnd-6_family-3886 HVAEP8:7054418-7055990
DNA/hAT-hAT5 rnd-5_family-1804 HVAEP1:80726820-80728065
DNA/TcMar-Tc1 rnd-6_family-5145 HVAEP1:77640853-77642229
DNA/CMC-EnSpm rnd-5_family-3934 HVAEP2:3057697-3059127
LTR/Gypsy rnd-6_family-9419 HVAEP1:55586576-55590596
DNA/CMC-EnSpm rnd-5_family-3934 HVAEP2:15005210-15007293
DNA/TcMar-Tc1 rnd-6_family-5145 HVAEP1:78976511-78978355
DNA/TcMar-Fot1 rnd-5_family-253 HVAEP1:33060294-33063087
DNA/MULE-MuDR rnd-6_family-7159 HVAEP14:4120942-4123454
LTR/Gypsy rnd-1_family-1419 HVAEP10:43090724-43092658
DNA/hAT-Ac rnd-6_family-1056 HVAEP13:10362962-10370312
DNA/TcMar-Tc1 rnd-6_family-5145 HVAEP15:54130926-54132562
DNA/CMC-EnSpm rnd-6_family-3074 HVAEP10:25963147-25969663
DNA/TcMar-Tc1 rnd-6_family-5145 HVAEP15:15536-17367
DNA/TcMar-Tc1 rnd-6_family-5145 HVAEP8:384659-386495
DNA/TcMar-Tc1 rnd-6_family-5145 HVAEP15:668768-670443
DNA/CMC-EnSpm rnd-5_family-3934 HVAEP2:4751649-4753120
DNA/hAT-Ac rnd-6_family-1056 HVAEP13:10921738-10928448
DNA/PIF-Harbinger rnd-1_family-55 HVAEP6:59917008-59919706
DNA/PIF-Harbinger rnd-1_family-55 HVAEP2:14396561-14399263
DNA/PIF-Harbinger rnd-1_family-55 HVAEP2:39514731-39517434
DNA/CMC-EnSpm rnd-5_family-3934 HVAEP2:6790473-6791835
DNA/CMC-EnSpm rnd-5_family-3934 HVAEP2:9510292-9511759
LINE/Rex-Babar rnd-6_family-3886 HVAEP8:8897684-8899249
DNA/Zisupton rnd-6_family-8870 HVAEP7:9662698-9666983
DNA/TcMar-Tc1 rnd-6_family-5145 HVAEP6:61221861-61223122
DNA/hAT-hAT5 rnd-5_family-1804 HVAEP1:27370425-27371852
DNA/CMC-EnSpm rnd-6_family-8718 HVAEP4:51715338-51716393
DNA/TcMar-Tc1 rnd-6_family-5145 HVAEP6:61014250-61015933
DNA/PiggyBac rnd-5_family-10 HVAEP12:35582205-35583698
DNA/Maverick rnd-5_family-1623 HVAEP3:58847422-58852710
LINE/Rex-Babar rnd-6_family-3886 HVAEP8:9085704-9087260
DNA/TcMar-Tc1 rnd-6_family-5145 HVAEP6:60887710-60889349
DNA/TcMar-Tc1 rnd-6_family-5145 HVAEP15:54802215-54804058
DNA/CMC-EnSpm rnd-5_family-123 HVAEP8:11488555-11489810
DNA/hAT-hAT5 rnd-5_family-1804 HVAEP2:1282996-1284155
DNA/CMC-EnSpm rnd-5_family-3934 HVAEP1:27367624-27369080
DNA/CMC-EnSpm rnd-5_family-123 HVAEP6:10641717-10643981
Ec_BodyCol/SC
Ec_differentiated
En_BodyCol/SC
En_differentiated
I_ISC
I_differentiated
Ec_1
Ec_2
Ec_3
En_1
En_2
En_3
I_ISC_1
I_ISC_2
I_ISC_3
I_ISC_4
Percent expressed
0 10 20 30 40 50
Average expression
-1 0 1 2
Normalized expression
-4 -2 0 2 4

Fig. S6. Expression profiles of TEs at locus level

Expression profiles of TEs at locus level were investigated using two different datasets[3,4]. In each group of ectodermal stem cells (Ec_BodyCol/SC), endodermal stem cells (En_BodyCol/SC), and i-cells (I_ISC), top20 TEs that were significantly more expressed are shown. Average expression levels (color intensity) and the proportion of cells expressing them (circle size) are shown. Additionally, as a validation, the expression levels of these TEs in a FACS-sorted bulk RNA-seq dataset are displayed on a heatmap to the right.

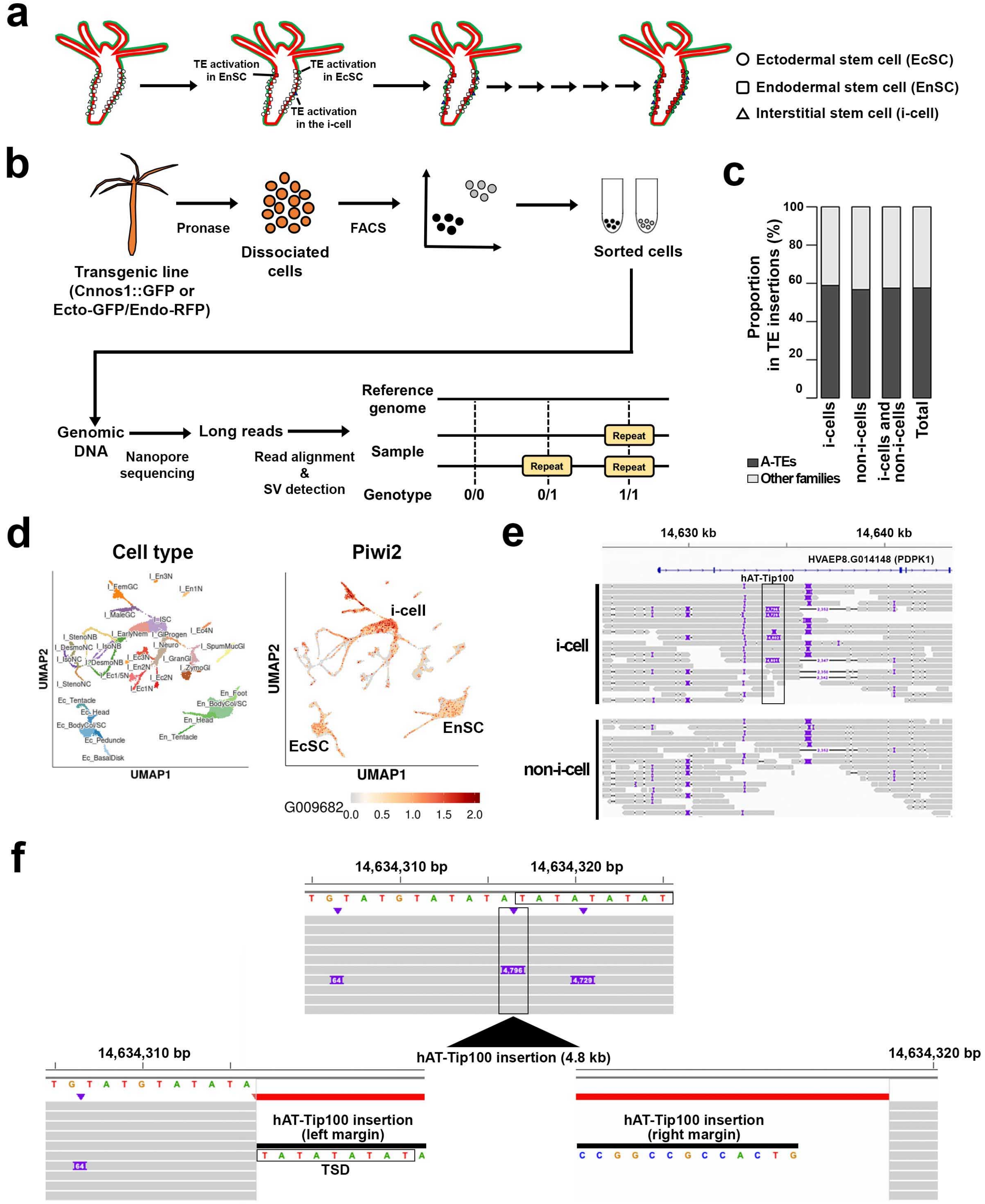

Fig. S7. Stem cell-lineage specific whole genome sequencing

(a) TE activation and stem cell genome divergence. **(b)** Workflow for stem cell-lineage specific whole genome sequencing. To elucidate the TE landscapes within the genomes of three stem cell lineages in hydra, transgenic lines expressing reporter genes (Cnnos1::GFP[5] or Ecto-GFP/Endo-RFP[6]) were clonally propagated. The hydra specimens were dissociated into single cells through

Pronase treatment, and the stem cell lineages were sorted based on fluorescence intensities using FACS. Genomic DNA was then extracted from the sorted cells, and long reads were obtained via Nanopore sequencing. The reads were mapped to the AEP genome sequence to identify insertion sites. The analysis focused on insertions longer than 1kb, searching for insertions with sequences homologous to a custom repeat library generated by RepeatModeler from the genome sequence (Methods). This approach allowed for the determination of the extent to which each TE subfamily is inserted into the genome across different cell populations, facilitating the analysis of TE-induced stem cell genome divergence through comparisons across cell populations. **(c)** Contribution of the A-TEs to the TE insertions. **(d)** Piwi2 expression in the stem cell. Piwi2 (G009682) is expressed in the all three stem cell populations (i-cell, EcSC, and EnSC). Panels were retrieved from the Hydra AEP Genome Project Portal (https://research.nhgri.nih.gov/HydraAEP/). **(e)** The i-cell specific hAT-Tip100 insertion in the intron of 3-phosphoinositide-dependent protein kinase 1 (PDPK1) involved in cell proliferation[7,8]. **(f)** Target site duplication of the the i-cell specific hAT-Tip100 insertion.

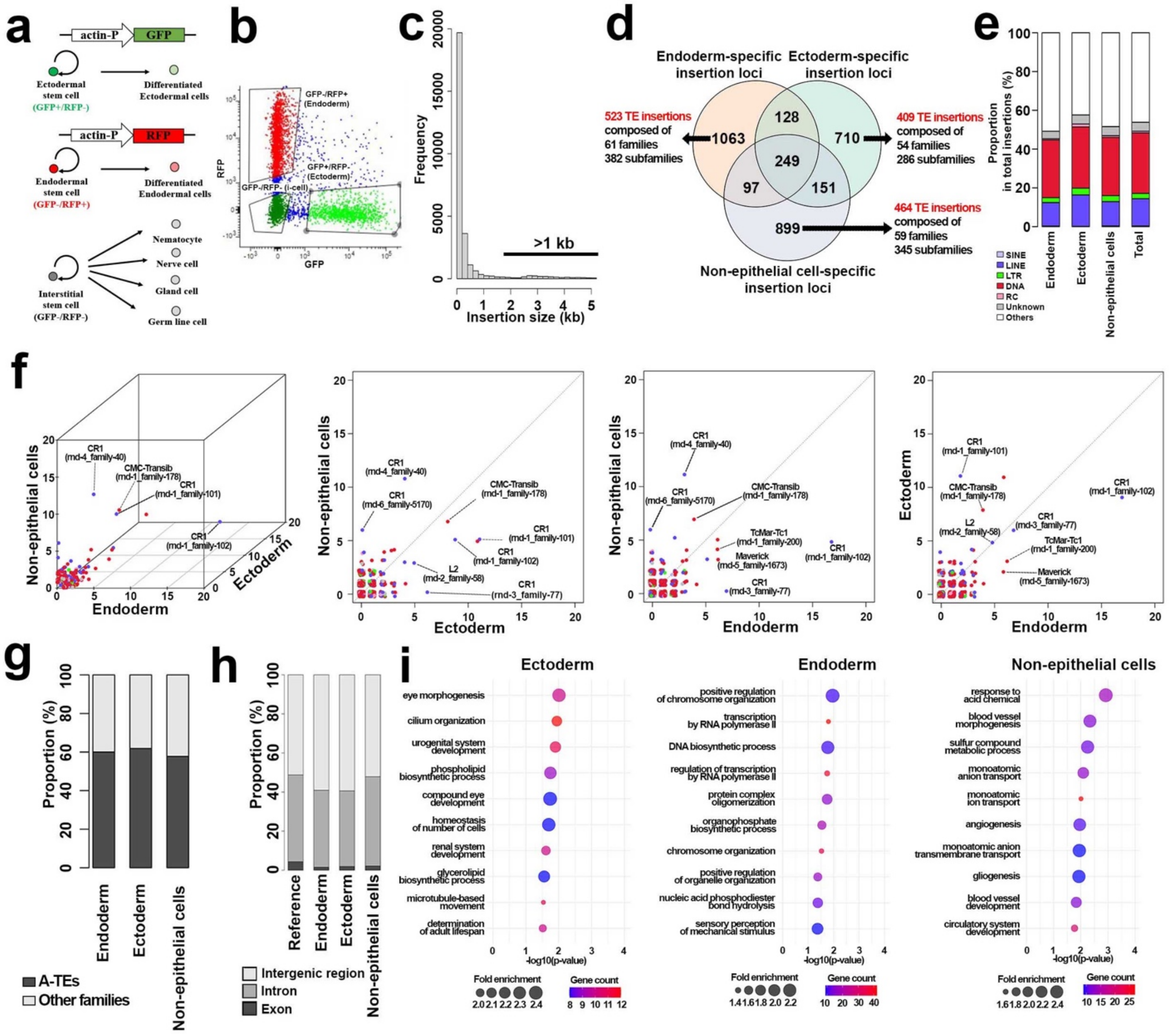

Fig. S8. TE insertion dynamics of stem cells in ecto-GFP/endo-RFP hydra

(a) ecto-GFP/endo-RFP transgenic line labelling the epithelial cells. **(b)** FACS-sorting of the ecotodermal cell population, endodermal cell population and non-epithelial cell population. **(c)** Size distribution of insertions. **(d)** Number of cell type specific and shared insertions. **(e)** Major types of TEs contributing to the insertions. **(f)** Counts of TE subfamilies in the TE insertions at the specific loci for each population. **(g)** Contribution of the A-TEs to the insertions. **(h)** Distributions of insertions identified in intergenic regions, introns, and exons. **(i)** GO-term enrichment analysis of closest genes to the insertions detected.

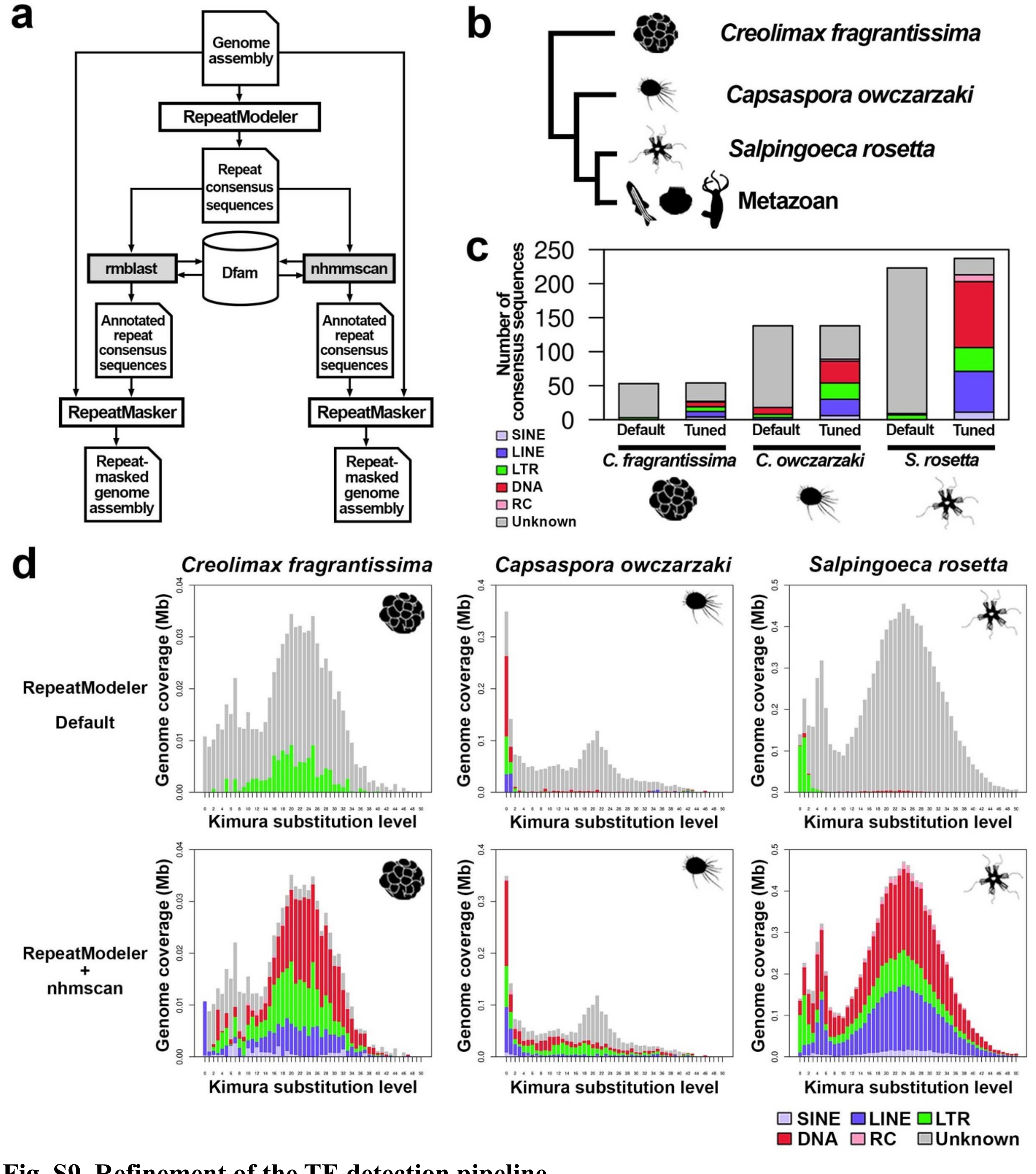

Fig. S9. Refinement of the TE detection pipeline
(a) Flowchart of the pipeline for detecting TEs. **(b)** Cladogram of species used for the benchmark analysis. **(c)** Annotation of repeat consensus sequences generated by RepeatModeler. (d) Changes in the repeat landscape.

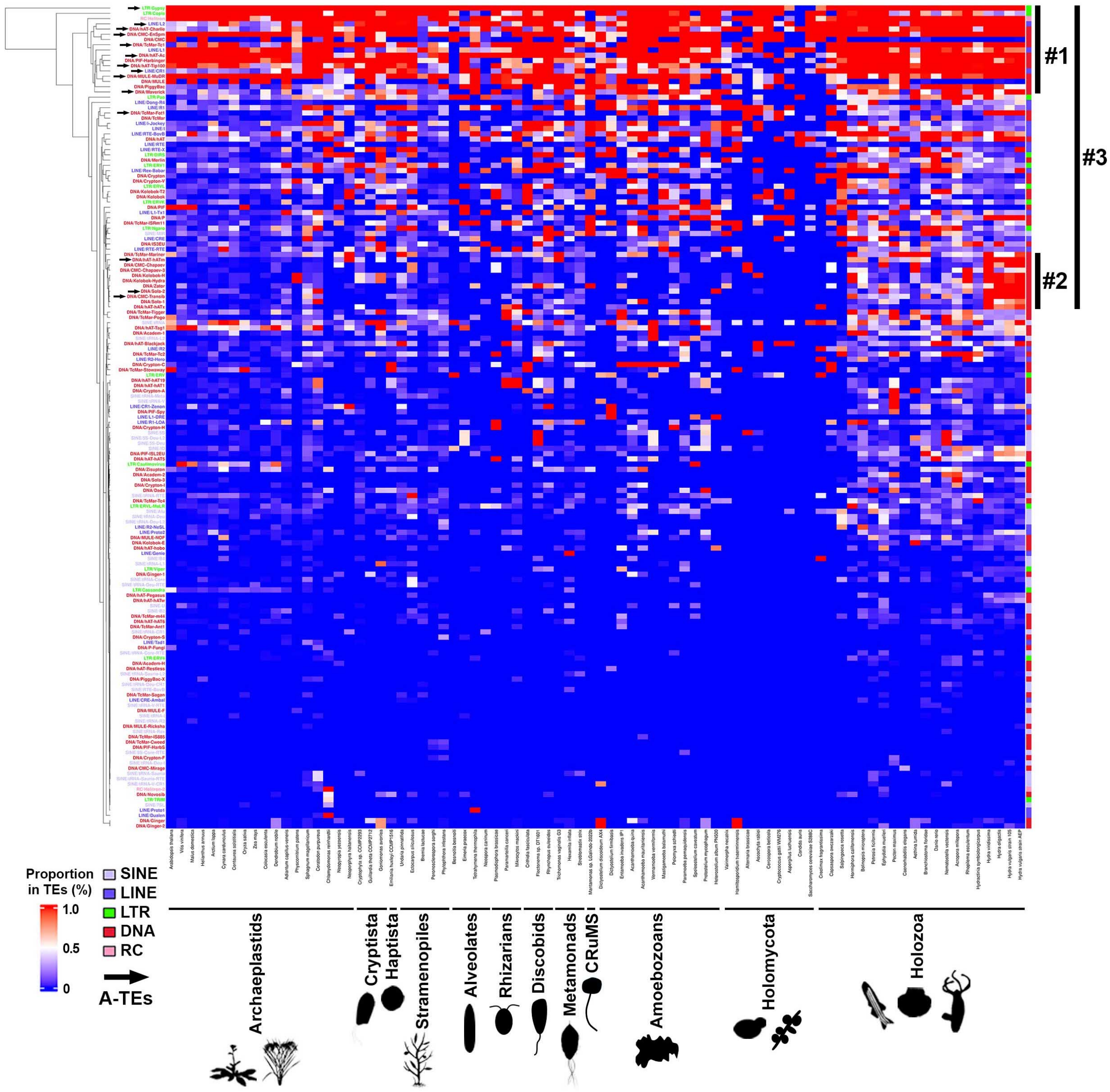

Fig. S10. Deep TE family homology across eukaryotes (related to Fig. 5)
Compositions of TEs in 82 eukaryote genomes. The heatmap represents the extent to which the genome coverage of each TE family constitutes the overall genome coverage of TEs for each species. The dendrogram at the left of the heatmap represents the result of hierarchical clustering performed using the Euclidean distance metric and the Ward's method. TEs in the cluster #3 are highlighted in Fig. 5.

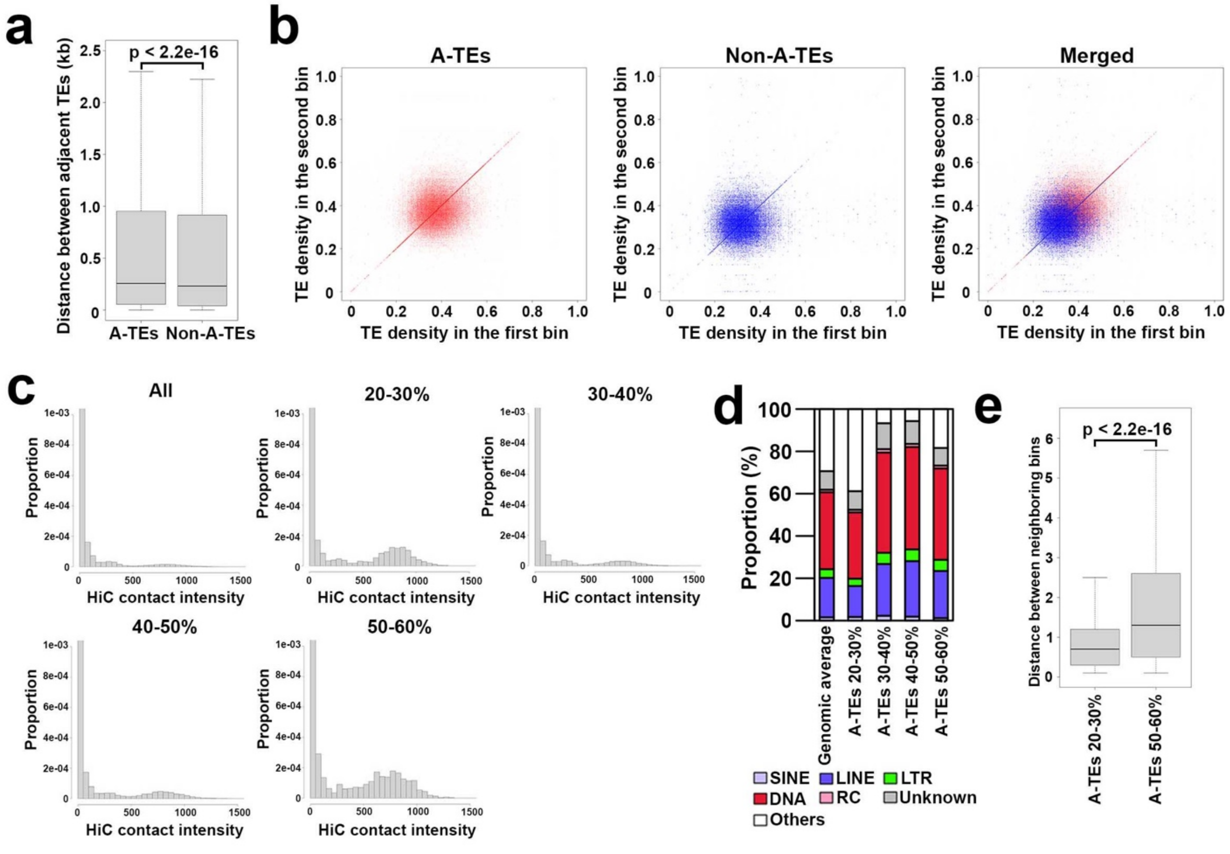

Fig. S11. A-TEs and chromosomal conformation

(a) The distance between adjacent A-TEs and between adjacent non-A-TEs. **(b)** TE density and chromatin contact intensity. Both axes represent TE density in bins, while chromatin contact intensities are indicated by the color gradients. **(c)** Relationship between A-TE coverage in bins and chromatin contact intensity. **(d)** Average TE coverage across bins. **(e)** The relationship between A-TEs coverage within a bin and the distance between bins. For bins with 20-30% A-TEs and bins with 50-60% A-TEs, the distances between bins were measured by extracting only bins with Hi-C contact intensity of 500-1000.

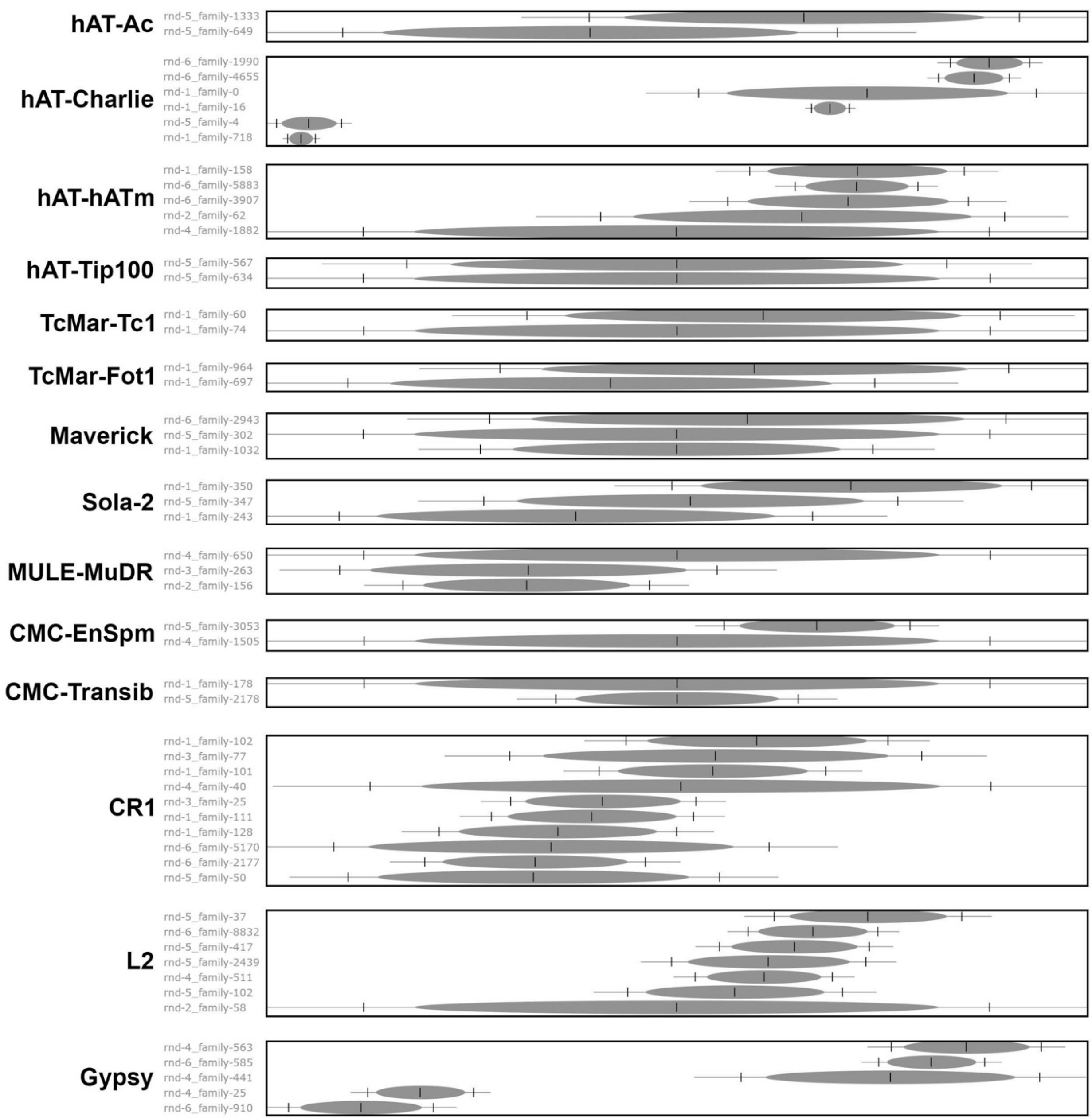

Fig. S12. Nested insertion patterns of A-TEs

Nested insertion patterns are shown for each A-TE family, and for each of its comprising RepeatModeler subfamilies (consensus sequences). Relative TinT timescale: old elements (left, diverse TinTs), young elements (right, less TinTs). The center of the ovals represents the maximum of each activity period, the end of the oval indicates 75%, and the vertical outer lines represent 99% of the probable activity period.

Supplementary Note 1. Brief summary of TE evolution and function, and the context to this study

Interspersed repetitive elements have long been studied in animal genome evolution and beyond[9–11]. Specifically for animals, the sequencing of the first "non-model" genomes have revealed a "complex" repeat repertoire[2,12], comprised not only of simple repeats, but also many representatives of major DNA and Retroelement classes[13]. Each of these studied have identified hundreds to thousands of repetitive element families, many of which were found to be expanded in a species-specific pattern.

The function of these repeats has been discussed along several research lines. First, the most obvious result of the accumulation of repetitive elements is the increase in the genome size. Many studies have reported how increase in several[14,15] or single[16] repeat families or classes can result in several-fold increase in the genome size. Some of the most striking observation was the repeat expansions present in salamanders, lungfishes[17–19], other vertebrates[20], and plants[21]. Interestingly, as several studies have found, such expansions do not play a major role in large-scale genome rearrangements and the ancestral linkage groups are preserved[1,18]. This is also evident in species that have prevailed at repetitive element expulsion, and these species often show highly rearranged genomes[12,22,23]. Nevertheless, repeat elements, when accumulated in hotspots, can cause enhanced recombination, in particular in mammals[24,25].

The role of repeats in generating novel regulatory elements has been explored in many systems[26], most functional data available for mammals[27]. Insertion of repeats can affect gene expression and adaptation[28,29], as well as drive formation of putative enhancers and other regulatory regions[30,31]. Certain repeat classes and satellite repeats have been shown to facilitate duplications of particular gene families, such as zinc-fingers[32,33] or long noncoding RNAs (lncRNAs)[34]. More recently, a more direct role of protein products from certain retro-elements during fish development[35] or transcription factor evolution[36]. Retro-elements have also been recently implicated in structural folding of chromatin[37].

The functional role of somatic insertions of repeat elements has been less investigated beyond model species. In *Drosophila*, structural variant accumulation in neuronal genomes has been debated[38] while general expression of repeats in various organisms has been widely reported[25,39–41].

These data paint a very diverse and often anecdotal picture of repeat evolution and function that, due to the large numbers of repeats and their loci and their complex evolutionary relationships, makes it a daunting task to identify general principles of TE accumulation in metazoan genomes. To this end, the biggest missing point has been the lack of a comparative and anciently branching model where TE activity can be dissected at single-cell transcriptional and genomic levels.

Supplementary Note 2. TE cross-validation and subfamily-level quantification

For cross-validation of the TE annotation in the current study, we evaluated the consistency of the TE annotation in this study with other database sources[42,43]. We found that the masked genomic regions in the genome using the custom repeat library from the current study generally overlapped

with regions annotated as corresponding repeat families in RepBase[42] library (Additional file 2: Table S12). For example, genomic regions marked as LTR/Gypsy superfamily using our custom repetitive element library were found to correspond to hundreds of known Gypsy families in the RepBase. However, only 8 of these families (out of 27,549 Gypsy sequences) occupied more than 50% of all Gypsy genomic regions based on the RepBase (Additional file 2: Table S13). Similarly, in the dataset of TE insertions in stem cell lineages, out of 162 Gypsy insertions (based on our Dfam-based analysis), 155 (95.7%) corresponded to 124 Gypsy sequences in Repbase, with Gypsy-19_HM-I being the most common. (Additional file 2: Table S14). Similar classification was reached using GypsyDB[43] as reference (Additional file 2: Table S15). These results indicate a notable consistency between the TE annotations in this study and established database resources.

In addition to the family-level quantification outlined in the main text, this section also describes subfamily (consensus sequence) level analysis. Overall similar trends were observed between family and subfamily levels. Due to their higher numbers subfamilies of each A-TE family from show a more diverse size distribution between the AEP and 105 genomes (Additional file 1: Fig. S3). For example, only few TcMar-Tc1 and CR1 subfamilies were enriched in the AEP genome while the majority of hAT-Ac and Gypsy subfamilies had a contribution to the AEP genome size increase (Additional file 1: Fig. S3).

At the subfamily level, expression of 868 subfamilies and 762 subfamilies were observed from the AEP and the 105 haplotype, respectively. TE subfamily coverage in the Iso-seq reads of the AEP strain was 59%±21 (mean±standard deviation), and that of the 105 was 57%±27 suggesting that more than half of regions of each TE-derived read is derived from a TE on average (Additional file 1: Fig. S5e). At the subfamily level, 44 types of TEs showed cell-type-specific expression profiles, and at the loci-level analysis, 203 types of TEs exhibited cell-type-specific expression profiles (Fig. 3e, Additional file 1: Fig. S6, Additional file 2: Table S6,7). The expression profiles measured by scRNA-seq were also supported by FACS-sorted bulk RNA-seq datasets[4] (Additional file 1: Fig. S6, Methods).

In respect to insertions, 322 TE subfamilies were detected in the i-cell population; the non-i-cell population insertions was associated with 200 TE subfamilies; and the shared insertions contained 1,013 TE subfamilies (Fig. 4d). Of the TE insertions identified, 58.9% were in the i-cell population, 56.7% in the non-i-cell population, and 57.5% in those found in both the i-cell and non-i-cell populations were attributed to the A-TEs suggesting the high activities of the A-TEs in each of the stem cell lineage (Additional file 1: Fig. S7c). In both the i-cell and non-i-cell populations, the CR1 (rnd-1_family-102) and TcMar-Tc1 (rnd-1_family-200) subfamilies were the most prevalent TE subfamily insertions (Fig. 4g). In the scRNA-seq datasets of the hydra polyps[3], TE subfamilies, which showed genomic insertions in the stem-cell lineage, showed relatively broad expression suggesting underlying potential contributions of chromatin states to the TE insertion preference (Fig. 4h). Furthermore, in the ecto-GFP/endo-RFP transgenic line, we identified 286 subfamilies in ectodermal TE insertions, 382 subfamilies in endodermal TE insertions, and 345 subfamilies in non-epithelial cell populations (Additional file 1: Fig. S8c,d).

References

1. Simakov, O. *et al.* Deeply conserved synteny and the evolution of metazoan chromosomes. *Sci. Adv.* **8**, eabi5884 (2022).
2. Chapman, J. A. *et al.* The dynamic genome of Hydra. *Nature* **464**, 592–6 (2010).
3. Siebert, S. *et al.* Stem cell differentiation trajectories in Hydra resolved at single-cell resolution. *Science* **365**, (2019).
4. Nishimiya-Fujisawa, C. *et al.* An ancient split of germline and somatic stem cell lineages in Hydra. *bioRxiv* (2023).
5. Nishimiya-Fujisawa, C. & Kobayashi, S. Germline stem cells and sex determination in Hydra. *Int. J. Dev. Biol.* **56**, 499–508 (2012).
6. Glauber, K. M. *et al.* A small molecule screen identifies a novel compound that induces a homeotic transformation in Hydra. *Development* **142**, 2081 (2015).
7. Gagliardi, P. A., di Blasio, L. & Primo, L. PDK1: A signaling hub for cell migration and tumor invasion. *Biochim. Biophys. Acta* **1856**, 178–88 (2015).
8. Raimondi, C. & Falasca, M. Targeting PDK1 in cancer. *Curr. Med. Chem.* **18**, 2763–9 (2011).
9. Feschotte, C. & Pritham, E. J. DNA transposons and the evolution of eukaryotic genomes. *Annu. Rev. Genet.* **41**, 331–68 (2007).
10. Feschotte, C. Transposable elements and the evolution of regulatory networks. *Nat. Rev. Genet.* **9**, 397–405 (2008).
11. Wells, J. N. & Feschotte, C. A Field Guide to Eukaryotic Transposable Elements. *Annu. Rev. Genet.* **54**, 539–561 (2020).
12. Putnam, N. H. *et al.* Sea Anemone Genome Reveals Ancestral Eumetazoan Gene Repertoire and Genomic Organization. *Science (80-.).* **317**, 86–94 (2007).
13. Bourque, G. *et al.* Ten things you should know about transposable elements. *Genome Biol.* **19**, 199 (2018).
14. Naville, M. *et al.* Massive Changes of Genome Size Driven by Expansions of Non-autonomous Transposable Elements. *Curr. Biol.* **29**, 1161-1168.e6 (2019).
15. Blommaert, J., Riss, S., Hecox-Lea, B., Mark Welch, D. B. & Stelzer, C. P. Small, but surprisingly repetitive genomes: transposon expansion and not polyploidy has driven a doubling in genome size in a metazoan species complex. *BMC Genomics* **20**, 466 (2019).
16. Wong, W. Y. *et al.* Expansion of a single transposable element family is associated with genome-size increase and radiation in the genus Hydra. *Proc. Natl. Acad. Sci. U. S. A.* **116**, 22915–22917 (2019).
17. Nowoshilow, S. *et al.* The axolotl genome and the evolution of key tissue formation regulators. *Nature* **554**, 50–55 (2018).
18. Meyer, A. *et al.* Giant lungfish genome elucidates the conquest of land by vertebrates. *Nature* **590**, 284–289 (2021).
19. Sun, C. & Mueller, R. L. Hellbender Genome Sequences Shed Light on Genomic Expansion at the Base of Crown Salamanders. *Genome Biol. Evol.* **6**, 1818–1829 (2014).
20. Kapusta, A., Suh, A. & Feschotte, C. Dynamics of genome size evolution in birds and mammals. *Proc. Natl. Acad. Sci.* **114**, (2017).
21. Tenaillon, M. I., Hollister, J. D. & Gaut, B. S. A triptych of the evolution of plant transposable elements. *Trends Plant Sci.* **15**, 471–478 (2010).
22. DeBarry, J. D. & Kissinger, J. C. Jumbled Genomes: Missing Apicomplexan Synteny. *Mol. Biol. Evol.* **28**, 2855–2871 (2011).

23. Kissinger, J. C. & DeBarry, J. Genome cartography: charting the apicomplexan genome. *Trends Parasitol.* **27**, 345–354 (2011).
24. McVean, G. What drives recombination hotspots to repeat DNA in humans? *Philos. Trans. R. Soc. B Biol. Sci.* **365**, 1213–1218 (2010).
25. O'Neill, R. J. W., O'Neill, M. J. & Graves, J. A. M. Undermethylation associated with retroelement activation and chromosome remodelling in an interspecific mammalian hybrid. *Nature* **393**, 68–72 (1998).
26. Chuong, E. B., Elde, N. C. & Feschotte, C. Regulatory activities of transposable elements: from conflicts to benefits. *Nat. Rev. Genet.* **18**, 71–86 (2017).
27. Emera, D. & Wagner, G. P. Transformation of a transposon into a derived prolactin promoter with function during human pregnancy. *Proc. Natl. Acad. Sci.* **109**, 11246–11251 (2012).
28. Papolu, P. K. *et al.* Retrotransposons: How the continuous evolutionary front shapes plant genomes for response to heat stress. *Front. Plant Sci.* **13**, (2022).
29. Gianfrancesco, O., Bubb, V. J. & Quinn, J. P. SVA retrotransposons as potential modulators of neuropeptide gene expression. *Neuropeptides* **64**, 3–7 (2017).
30. Du, A. Y., Chobirko, J. D., Zhuo, X., Feschotte, C. & Wang, T. Regulatory transposable elements in the encyclopedia of DNA elements. *Nat. Commun.* **15**, 7594 (2024).
31. Fueyo, R., Judd, J., Feschotte, C. & Wysocka, J. Roles of transposable elements in the regulation of mammalian transcription. *Nat. Rev. Mol. Cell Biol.* **23**, 481–497 (2022).
32. Wells, J. N. *et al.* Transposable elements drive the evolution of metazoan zinc finger genes. *Genome Res.* **33**, 1325–1339 (2023).
33. Eichler, E. E. *et al.* Complex β-Satellite Repeat Structures and the Expansion of the Zinc Finger Gene Cluster in 19p12. *Genome Res.* **8**, 791–808 (1998).
34. Kapusta, A. *et al.* Transposable Elements Are Major Contributors to the Origin, Diversification, and Regulation of Vertebrate Long Noncoding RNAs. *PLoS Genet.* **9**, e1003470 (2013).
35. Chang, N.-C. *et al.* Gag proteins encoded by endogenous retroviruses are required for zebrafish development. *bioRxiv* **10.1101/20**, (2024).
36. Cosby, R. L. *et al.* Recurrent evolution of vertebrate transcription factors by transposase capture. *Science (80-.).* **371**, (2021).
37. Lu, J. Y. *et al.* Homotypic clustering of L1 and B1/Alu repeats compartmentalizes the 3D genome. *Cell Res.* **31**, 613–630 (2021).
38. Li, W. *et al.* Activation of transposable elements during aging and neuronal decline in Drosophila. *Nat. Neurosci.* **16**, 529–531 (2013).
39. Petersen, H. O. *et al.* A Comprehensive Transcriptomic and Proteomic Analysis of Hydra Head Regeneration. *Mol. Biol. Evol.* **32**, 1928–47 (2015).
40. Albertin, C. B. *et al.* The octopus genome and the evolution of cephalopod neural and morphological novelties. *Nature* **524**, 220–224 (2015).
41. Dion-Côté, A.-M., Renaut, S., Normandeau, E. & Bernatchez, L. RNA-seq Reveals Transcriptomic Shock Involving Transposable Elements Reactivation in Hybrids of Young Lake Whitefish Species. *Mol. Biol. Evol.* **31**, 1188–1199 (2014).
42. Bao, W., Kojima, K. K. & Kohany, O. Repbase Update, a database of repetitive elements in eukaryotic genomes. *Mob. DNA* **6**, 11 (2015).
43. Llorens, C. *et al.* The Gypsy Database (GyDB) of mobile genetic elements: release 2.0. *Nucleic Acids Res.* **39**, D70–D74 (2011).
